# Swallowing Exercise During Head and Neck Cancer Treatment: Results of a Randomized Trial

**DOI:** 10.1007/s00455-021-10320-5

**Published:** 2021-06-11

**Authors:** Sara Fredslund Hajdú, Irene Wessel, Susanne Oksbjerg Dalton, Signe Janum Eskildsen, Christoffer Johansen

**Affiliations:** 1 Department of Occupational Therapy and Physiotherapy, Copenhagen University Hospital Rigshospitalet, Esther Møllers vej 8, 2100 Copenhagen, Denmark; 2 Cancer Late Effects Research Unit (CASTLE), Department of Oncology, Copenhagen University Hospital Rigshospitalet, Blegdamsvej 9, 2100 Copenhagen, Denmark; 3 Department of Otorhinolaryngology, Head and Neck Surgery & Audiology, Copenhagen University Hospital Rigshospitalet, Blegdamsvej 9, Copenhagen, Denmark; 4 Danish Research Centre for Equality in Cancer (COMPAS), Zealand University Hospital, Ringstedgade 61, 4700 Naestved, Denmark; 5 Survivorship and Inequality in Cancer, Danish Cancer Society Research Centre, Strandboulevarden 49, 2100 Copenhagen, Denmark

**Keywords:** Deglutition disorders, Head and neck neoplasms, Rehabilitation, Exercise

## Abstract

The diagnosis and treatment of head and neck cancer (HNC) can have substantial impact on swallowing function, nutritional balance, physical function and quality of life (QoL). Early initiated swallowing exercises are hypothesized to improve swallowing function in HNC patients. The aim was to investigate the effects of swallowing exercises and progressive resistance training (PRT) during radiotherapy on swallowing function, physical function and QoL in patients with pharynx-, larynx-, oral cavity cancer or unknown primary compared to usual care. In a multi-centre RCT participants were assigned to (a) twice-weekly PRT and daily swallowing exercises throughout treatment or (b) usual care. Outcomes were measured at end of treatment and 2, 6 and 12 months after. Primary outcome was penetration aspiration score (PAS). Data were analysed on an “intention-to-treat” basis by GEE logistic regression model, linear mixed effects model and cox regression. Of 371 invited HNC patients, 240 (65%) enrolled. Five participants were excluded. At 12 months follow-up, 59 (25%) participants were lost. Analyses showed significant effect on mouth opening, QoL, depression and anxiety at 12 months when comparing intervention to non-active controls. The trial found no effect on swallowing safety in HNC undergoing radiotherapy, but several positive effects were found on secondary outcomes when comparing to non-active controls. The intervention period may have been too short, and the real difference between groups is too small. Nevertheless, the need to identify long-lasting intervention to slow down or avoid functional deteriorations is ever more crucial as the surviving HNC population is growing.

**Supplementary Information:**

The online version contains supplementary material available at 10.1007/s00455-021-10320-5.

## Introduction

Head and neck cancer (HNC) can have substantial impact on swallowing function, nutritional balance, physical function and health related quality of life (HRQOL) [1, 2]. In a Danish randomized controlled trial (RCT) of 1476 patients with head and neck squamous cell carcinoma (HNSCC) eligible for primary radiotherapy, the prevalence of acute dysphagia was 83%. Chronic dysphagia prevalence declined to 46% and 23%, respectively, one and five years after treatment [3]. In a prospective cohort study of 425 Dutch HNC survivors [4], swallowing impairment and xerostomia was negatively associated with HRQOL. Further, based on data from a controlled intervention study of 266 HNC survivors, Daugaard et al. [5] found similar association between dysphagia and HRQOL. Dysphagia may result from multiple factors such as xerostomia, taste loss, impaired dental status, decreased sensory function, fibrosis and trismus [6–8]. Disuse of swallowing muscles can contribute to functional decline [9, 10]. Early initiated exercises that target the swallowing structures are hypothesized to counteract the effect of reduced spontaneous swallowing. However, studies examining the effect of preventive exercises in HNC patients are characterized by small RCTs [9, 11–15], or observational studies [10, 16–19].

A recent Cochrane review including 6 RCTs and 326 participants in total with advanced-stage HNC evaluated the effect of swallowing exercises on post-treatment swallowing function [20]. The authors found no effect of exercises before, during or immediately after radiotherapy, and stressed the lack of uniformity across trials making comparison difficult.

Furthermore, loss of lean body mass (LBM) in HNC patients has been associated with decreased physical performance despite stable energy intake [21]. Exercise that build muscle mass and -strength may be an important factor in maintaining performance level and nutritional status in HNC patients. The effect of progressive resistance training (PRT) on LBM was explored in a randomized trial including 41 patients with HNSCC [22]. Participants were randomly assigned to an early exercise or a delayed exercise group after radiotherapy. LBM increased by more than 4% in both groups, which was significantly more than after self-chosen activity [22].

With the aim to support the general health of HNC patients and minimize adverse late effects we conducted an RCT that examined the effect of a bimodal intervention with swallowing exercises and PRT performed during radiotherapy. Contrary to the RCTs published so far, we found a potential for investigating swallowing exercises in HNC including a larger sample and with an non-active control group, thus strengthening the differentiation between groups. Upon initiating the trial usual care did not include any type of swallowing therapy during radiotherapy. Individually designed swallow interventions rather than a ‘one size fits all’ model, and the combination of swallow exercise and PRT to preserve bodyweight and energy level, is unprecedented in clinical HNC trials. Primary endpoint was swallowing safety measured by degree of laryngeal penetration or aspiration one year after treatment. Secondary endpoints were duration of tube dependence and functional swallowing, mouth opening, physical functioning, HRQOL, depression and anxiety in the first year after radiotherapy.

## Setting and Participants

In SYNK (Danish ethical approval ID H-2-2014-074; clinicaltrial.gov, NCT02385929) we included 240 HNC patients from two hospitals in eastern Denmark. Participants were recruited from May 11th, 2015 (Rigshospitalet) and March 22nd, 2016 (Næstved Hospital) until September 21st, 2018, with final follow-up in November 2019. Eligibility criteria included persons ≥ 18 years of age, diagnosed with cancer in oropharynx, hypopharynx, oral cavity, larynx or unknown primary (UPT) and eligible for curatively intended radiotherapy treatment. Recruitment and baseline testing took place prior to, or within the first week of, radiotherapy. Participants were allocated to intervention or usual care in a 1:1 ratio by computer, stratified by treatment centre, tumour-site and concomitant chemotherapy in blocks of eight. Group allocation was concealed to outcome assessors, and participants were informed not to reveal their allocation. The protocol was described in detail elsewhere [23].

## Intervention

**Fig. 1:**
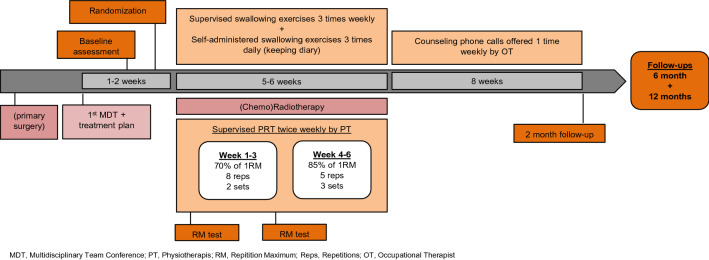
Study activities for the intervention group. *MDT* Multidisciplinary Team Conference; *PT* Physiotherapis; *RM* Repitition Maximum; *Reps* Repetitions; *OT* Occupational Therapist

The intervention group received usual care and the bimodal PRT and swallowing exercise intervention throughout radiotherapy. From the beginning of radiotherapy supervised swallowing exercise sessions by occupational therapist (OT) were scheduled three times weekly parallel with twice-weekly physiotherapy-led PRT. Self-administered swallowing exercises were prescribed based on individual assessments and were to be performed with up to 10 repetitions each, three times daily, 7 days per week during radiotherapy. Exercise programs consisted of all or some of the following 14 exercises: reaching tongue back and forth; tongue to cheek, tongue to mouth corners, resistance to tongue, gargle, yawn, mouth opening, jaw side-to-side, jaw undershot, Valsalva, Shaker exercise, Mendelsohn maneuver, Masako maneuver, Effortful swallow. Planning of the exercise programs was based on an individual assessment taking patient motivation, mental surplus, side effects, symptoms and t-site into consideration. A detailed swallowing exercise protocol can be found in online resource 1. Participants logged their home-training until end-of-treatment. In the following two months participants were offered weekly counselling by phone and encouraged to continue swallow exercises. The swallowing intervention is described in detail elsewhere [24]. The PRT program involved 6 exercises covering lower limbs, upper body and core in a fixed progression model based on repetition maximum. Figure 1 shows a timeline and overview of the intervention.

## Usual Care

Usual care differed between the two treatment centres. At one centre control group did not receive any intervention (non-active control group). At the other centre all HNC patients were offered an individually tailored exercise plan at beginning of radiotherapy with regular OT follow-up averaging to every other week until 2 weeks after end-of-treatment (active control group). It is common practice in Denmark that dysphagia is within the expertise of an OT. All participants could participate in municipality-based rehabilitation services, commonly initiated after the 2-month follow-up.

## Outcome Assessment

**Fig. 2:**
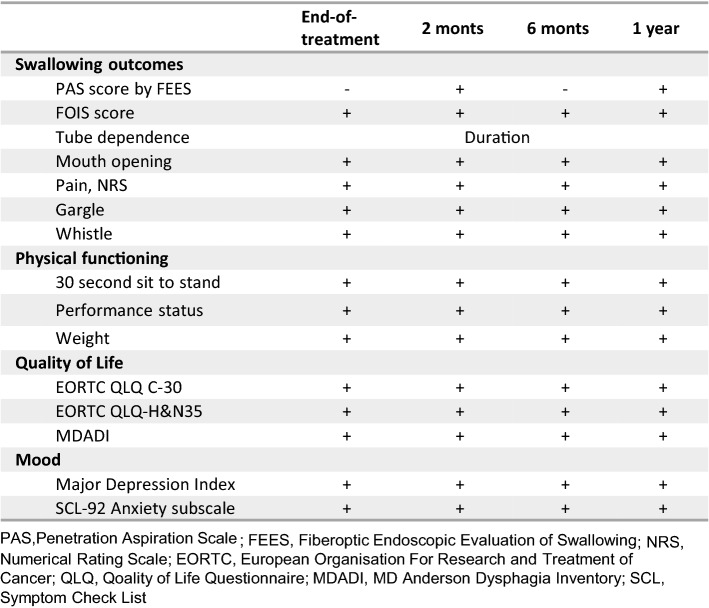
Outcome measures and time points of measurement. *PAS* penetration aspiration scale; *FEES* fiberoptic endoscopic evaluation of swallowing; *NRS* numerical rating scale; *EORTC* European Organisation For Research and Treatment of Cancer; *QLQ* Qoality of Life Questionnaire; *MDADI* MD Anderson Dysphagia Inventory; *SCL* Symptom Check List

All outcomes were assessed at baseline, end-of-treatment and 2, 6 and 12 months after end-of-treatment except the primary outcome which was assessed at baseline, and 2 and 12 months after (Fig. 2). Swallowing safety was scored by penetration aspiration scale (PAS) ranging from 1 (normal function) to 8 (silent aspiration) on liquid and moderately thickened liquid (IDDSI level 3 [25]) based on a fiberoptic endoscopic evaluation of swallowing (FEES). As the FEES examination was the most burdensome outcome measure to the patients in terms of discomfort and time spent, and based on clinical experience, we did not find it feasible to perform FEES examination at end-of-treatment and 6 months follow-up. Post hoc analyses were made on pharyngeal residual in the vallecula and the piriform sinuses from all available FEES recordings and scored on the YALE pharyngeal residue severity rating scale [26]. Ratings were provided numeric qualities from 1 (no residue) to 5 (severe).

Physical strength was measured by 30 Second Sit-To-Stand; mouth opening as maximal interincisal distance (MID) by Therabite Range of Motion Scale; performance status by ECOG Scale of Performance Status and functional swallowing by the Functional Oral Intake Scale (FOIS). Gargle and whistle function were assessed by simple non-standardized tests and dichotomized as yes/no. Gargle test was performed by taking a cup-sip of water, tilting the head back, gargle the water in the back of the throat for a few seconds, putting head in an upright position again and swallow. It was recorded as ‘no’ if the patient coughed, i.e. showed signs of penetration or aspiration. HRQOL was assessed by the European Organization for Research and Treatment of Cancer QoL Core Questionnaire (EORTC QLQ C-30 version 3.0) [27], the head and neck module (EORTC QLQ-H&N35) [28], and MD Anderson Dysphagia Inventory (MDADI) [29]. Pain was additionally measured by self-reported numerical rating scale (NRS). Depression and anxiety were assessed by Major Depression Index (MDI) and Symptom Check List (SCL-92 Anxiety subscale), respectively.

The EORTC QLQ C-30 comprises a 2-item scale for overall QoL, 5 function scales (physical, role, emotional, cognitive and social function) and 9 symptom scales (fatigue, nausea & vomiting, pain, dyspnoea, insomnia, appetite loss, constipation, diarrhoea, financial difficulties). All scales range from 0 to 100 with a higher score representing better functional status, higher QoL or, for symptom scales, a higher level of symptom.

The EORTC QLQ-H&N35 comprises seven symptom scales (pain, swallowing, senses, speech, social eating, social contact and sexuality), and 11 single items (teeth, mouth opening, dry mouth, sticky saliva, coughing, feeling ill, pain killers, nutritional supplements, feeding tube, weight loss and -gain). Scores range from 0 to 100, and a higher score represents more severe problems.

MDADI consists of 20 questions related to swallowing and covers three sub-scales (emotional, functional and physical) and a general QoL item. Each is scored as a mean score and multiplied by 20. Scores range from 20 (low functioning/QoL) to 100 (high functioning/QoL).

MDI consists of 12 questions each scored from 0 to 5. Four questions cover two groups of which only the questions with the highest score is included, providing a total score from 0 to 50. A score below 20 is not considered depression.

The SCL-92 anxiety subscale consists of 10 items, each scored from 0 to 4 with a total mean score ranging from 0 (no anxiety) to 40 (high level of anxiety).

Demographical data were collected via baseline questionnaires. Detailed medical information and tube dependence were collected via medical records.

## Statistical Analyses

The study was powered to detect a 10+ points change on the EORTC fatigue subscale, considered a moderate to large clinically significant change. Assuming a significance level of 5% and power of 80%, allowing a 20% loss to follow-up, the estimated sample size was determined to be 240 patients.

Early study data on penetration and aspiration were based on small samples, long-term follow-up (> 5 years) [30] or on laryngeal cancers only [31]. Fatigue was considered the primary outcome for the PRT modality of the intervention. As fatigue is a widely used outcome in cancer exercise trials and it is well documented that exercise has a positive impact on fatigue levels [32–34] we based sample size calculation on fatigue.

Data were treated as repeated measurements with assessments at baseline and four follow-up points. For binary outcomes we used a GEE logistic regression model, and for the continuous outcomes we used a linear mixed effects model. All analyses were based on intention-to-treat models assuming that groups were similar at baseline, thus only differences at the follow-ups were investigated. PAS, mouth opening, and oral intake were analysed as both continuous and binary outcomes, considering normal vs abnormal. Both models were analysed on complete cases only and assume that data are missing at random. All analyses were adjusted for p16, sex, age group, marital status, tumour-site and chemo. Time to tube removal for patients with tube feeding was analysed with Cox regression model to take censoring into account. All statistical analyses were carried out using R version 3.6.1.

## Results

**Fig. 3:**
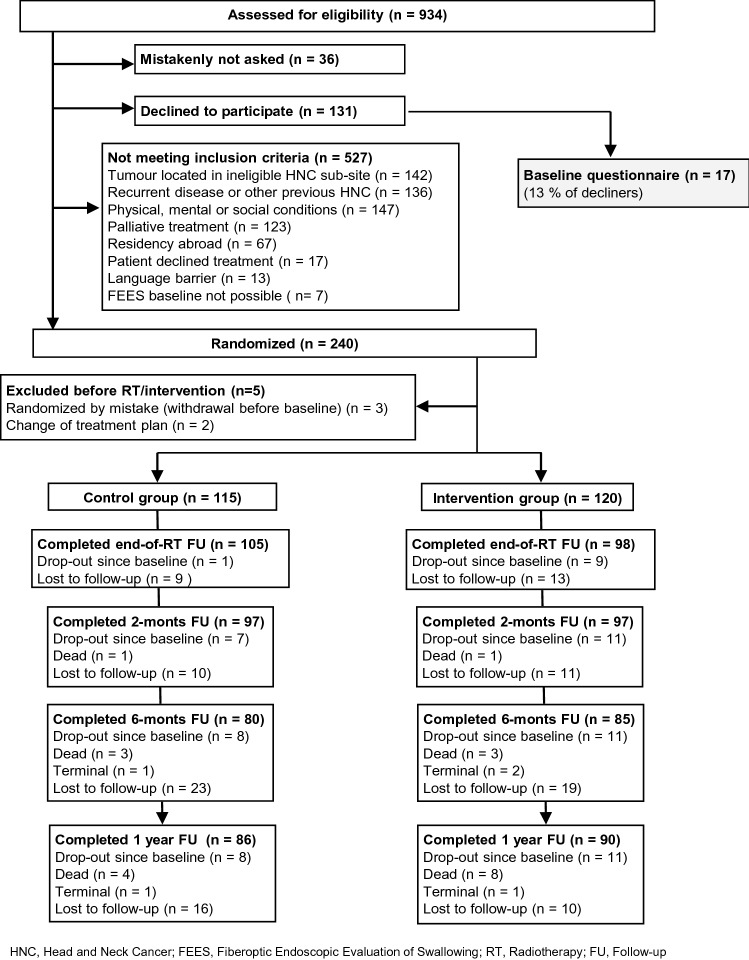
Flow diagram of enrolment and follow-up. *HNC* head and neck cancer; *FEES* fiberoptic endoscopic evaluation of swallowing; *RT* radiotherapy; *FU* follow-up

Of 371 invited HNC patients we enrolled 240 randomizing 118 to the control group and 122 to the intervention program. Three participants were randomized by mistake and another two were excluded due to change in treatment plan leaving a total 235 participants. Seven baseline questionnaires went missing. Nineteen participants (8%) had dropped out, 12 (5%) died and 2 (0.9%) were terminal at 12-month follow-up, and another 26 (11%) were lost to follow-up for other reasons. For the intervention group most dropouts occurred during intervention, mainly due to disliking the PRT. Figure 3 shows the flow of participants through the study.

Table 1 indicates a well-balanced randomization with equally distributed baseline characteristics except for total radiotherapy dose where the intervention group received less total Gy. Mean age at inclusion was 63 years [range 38–88]. Most participants (60%) had oropharynx cancer and received concurrent chemo-radiotherapy (54%). Eight percent had primary surgery. It appears as if non-participants (*n* = 17) are older and more likely to be female, p16-negative and current smoker and to have oral cancer and primary surgery, but differences were not tested for significance.

**Table 1:** Baseline characteristics of 235 head and neck cancer patients treated with (chemo)radiotherapy, SYNK trial, 2015–2018

Characteristic	Intervention *n* = 120	Control *n* = 115	*p*-value	NON-participants with baseline questionnaire (*n* = 17)
*Sociodemographic and physical profile*				
Mean age in years (SD)	63 (9)	63 (9)	*0.743*	66 (10)
Age below median (%)	60 (50)	53 (46)	*0.639*	8 (47)
Sex (%)			*0.310*	
Male	94 (78)	97 (84)		11 (65)
Female	26 (22)	18 (16)		6 (35)
Marital status (%)			*0.676*	
Single	30 (25)	32 (28)		7 (41)
In a relationship living alone	10 (8)	6 (5)		2 (12)
Cohabitating	76 (63)	71 (62)		8 (47)
Missing	4 (4)	6 (5)		–
Education^a^ (%)			*0.734*	
Low	25 (21)	22 (19)		6 (35)
Medium	35 (29)	39 (33)		9 (53)
High	60 (50)	54 (47)		2 (12)
*Disease and treatment information*				
Tumour-site (%)			*0.329*	
Oropharynx	68 (57)	72 (63)		5 (29)
Larynx	26 (22)	28 (24)		5 (29)
Oral cavity	10 (8)	5 (4)		5 (29)
Hypopharynx	11 (9)	9 (8)		2 (12)
Unknown primary	5 (4)	1 (1)		0
p16 (%)			*0.561*	
Positive	62 (52)	55 (48)		4 (24)
Negative	49 (41)	54 (47)		10 (59)
Unknown	9 (8)	6 (5)		3 (18)
Disease stage (%)			*0.458*	
I	27 (23)	28 (24)		5 (29
II	25 (21)	17 (15)		2 (12)
III	19 (16)	24 (21)		3 (18)
IVa	42 (35)	43 (38)		6 (35)
IVb	7 (6)	3 (3)		1 (6)
Treatment (%)			*0.546*	
Radiotherapy only	45 (38)	45 (39)		9 (53)
Concurrent chemo- and radiotherapy	63(53)	63 (55)		4 (24)
Surgery + (chemo)radiotherapy	12 (10)	7 (6)		4 (24)
Total GY (%)			***0***.***002***	
50	1 (1)	0		–
60	1 (1)	1 (1)		–
66	67 (56)	48 (42)		
68	43 (36)	66 (57)		
Unknown	8 (7)	0		–
*Health behaviour*				
Body mass index (%)			*0.242*	
< 18.5	5 (4)	2 (2)		–
18.5–25	44 (37)	44 (38)		–
> 25	68 (57)	69 (60)		–
Missing	3 (3)	–		17 (100)
Smoking			*0.913*	
Current	27 (23)	30 (26)		7 (41)
Used to	64 (54)	60 (52)		6 (35)
Never	25 (21)	21 (18)		3 (17)
Missing	4 (4)	4 (4)		1 (6)
Alcohol, units per week			*0.747*	
None	19 (16)	25 (22)		4 (24)
1–7	48 (40)	45 (39)		2 (12)
8–14	19 (16)	21 (18)		5 (29)
15–20	18 (15)	12 (10)		4 (24)
≥ 21	11 (9)	8 (7)		1 (6)
Missing	5 (4)	4 (4)		1 (6)

Bold italic values are indicate significant *p* value < 0.05

*SD* standard deviation

^a^Low education: primary through lower secondary, Medium education: Upper secondary, High education: Tertiary (> 13 years)

The overall results did not show differences between groups for the primary outcome (PAS), nor secondary outcomes with few exceptions. Participants in the intervention group had significantly better mouth opening (observed and experienced) and reported better social functioning, less pain on NRS, less anxiety, nausea & vomiting, appetite loss, constipation, and coughing at end-of-treatment, and less nausea and vomiting and senses problems 2 months after treatment. Conversely, the control group scored better on the MDADI functional domain at 2 and 6 months and had less pharyngeal residue 2 months after treatment (Table 2). The control group had better odds for normal gargle 1 year after treatment (online resource 2). Detailed tables of all binary outcomes can be found in online resource 2. Nasogastric tube placement was more frequent in the control group (55%) than in the intervention group (48%). Mean duration of tube dependence was 55 days (range 1–243) with no difference between groups (*p* = 0.63) (data not shown).

**Table 2:**
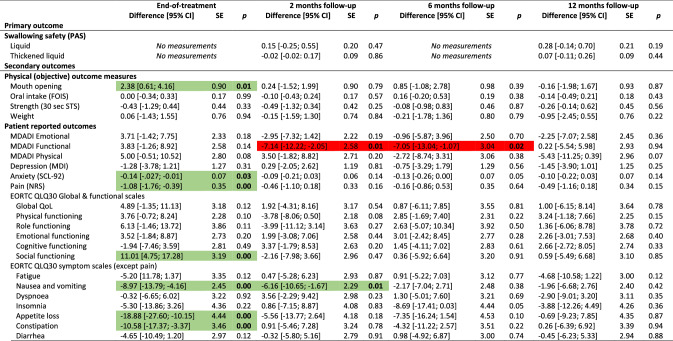

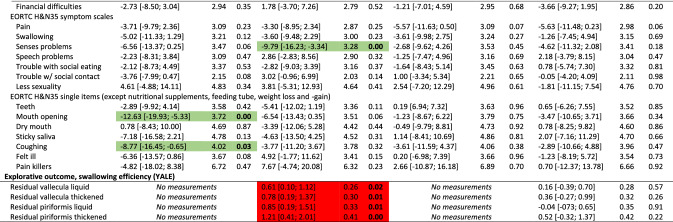
Effect of intervention at end-of-treatment and 2, 6 and 12 months after treatment: results of analyses of all continuous outcomes of 235 head and neck cancer patients, SYNK trial, 2015–2019, with adjustments for HPV (p16), sex, age group, marital status, tumour-site and chemo. Presenting differences in scores; intervention compared to control group^a^

*HPV* human papilloma virus, *CI* confidence interval, *SE* standard error, *PAS* penetration aspiration scale, *STS* Sit-to-Stand, *MDADI* MD Anderson Dysphagia Inventory, *SCL* Symptom Check List, *NRS* numerical rating scale, *EORTC* European Organization for Research and Cancer, *QlQ30* Quality of Life Questionnaire Core module, *H&N35* head and neck module

Green = significant in favour of intervention group, Red = significant in favour of control group

^a^Both active and non-active controls

Due to the differing control groups at the two hospitals, we performed post hoc analyses each hospital separately; one analysis on the hospital with the non-active control group (*n* = 128) and one analysis on the hospital with an active control group (*n* = 107). For the hospital with the non-active control group, more outcomes showed significant differences between groups and several with significant differences at 1-year follow-up in favour on intervention group for mouth opening, depression, anxiety, pain on NRS and EORTC H&N35, insomnia and use of pain killers (Table 3). Analysing the hospital with an active control group, fewer outcomes showed significant differences between groups compared to the analyses on the full sample. Some differences between intervention and control group remained, however, although mainly at end-of-treatment (Table 4).

**Table 3:**
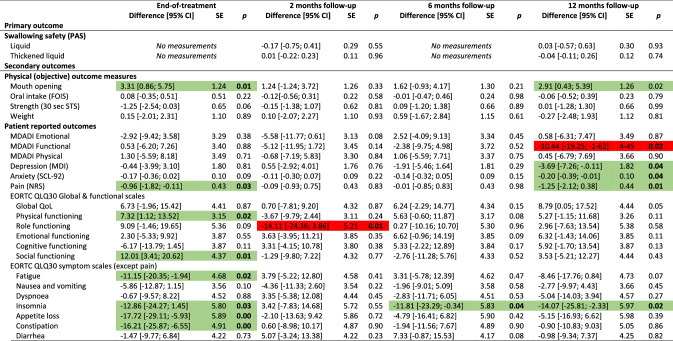

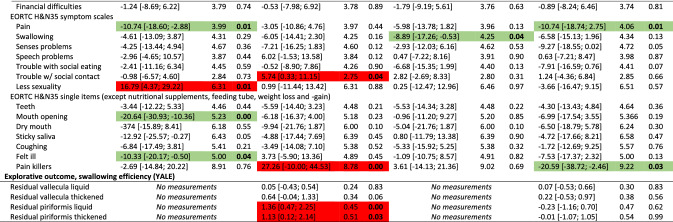
Effect of intervention at end-of-treatment and 2, 6 and 12 months after treatment: results of analyses of all continuous outcomes of 128 head and neck cancer patients, SYNK trial, 2015–2019, with adjustments for HPV (p16), sex, age group, marital status, tumour-site and chemo. Presenting differences in scores; intervention (*n* = 69) compared to non-active control (*n* = 59) group^a^

*HPV* human papilloma virus, *CI* confidence interval, *SE* standard error, *PAS* penetration aspiration scale, *STS* Sit-to-Stand, *MDADI* MD Anderson Dysphagia Inventory, *SCL* Symptom Check List, *NRS* numerical rating scale, *EORTC* European Organization for Research and Cancer, *QlQ30* Quality of Life Questionnaire Core module, *H&N35* head and neck module

Green = significant in favour of intervention group, Red = significant in favour of (non-active) control group

^a^Analysis based only on data from the hospital with a non-active control group

**Table 4:**
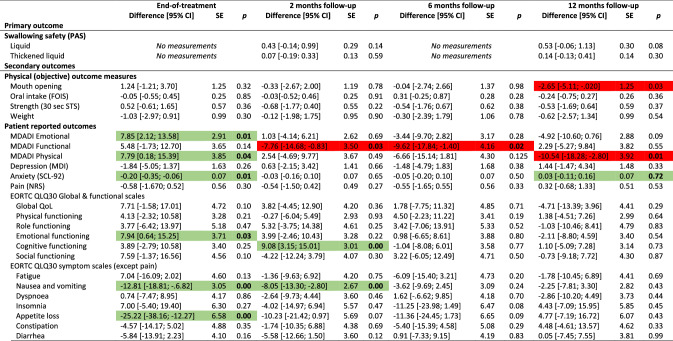

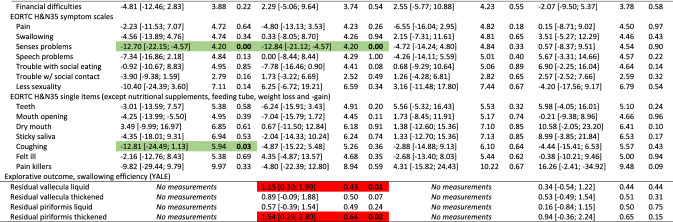
Effect of intervention at end-of-treatment and 2, 6 and 12 months after treatment: results of analyses of all continuous outcomes of 107 head and neck cancer patients, SYNK trial, 2016–2019, with adjustments for HPV (p16), sex, age group, marital status, tumour-site and chemo. Presenting differences in scores; intervention (*n* = 51) compared to active control (*n* = 56) group^a^

*HPV* human papilloma virus, *CI* confidence interval, *SE* standard error, *PAS* penetration aspiration scale, *STS* Sit-to-Stand, *MDADI* MD Anderson Dysphagia Inventory, *SCL* Symptom Check List, *NRS* numerical rating scale, *EORTC* European Organization for Research and Cancer, *QlQ30* Quality of Life Questionnaire Core module, *H&N35* head and neck module

Green = significant in favour of intervention group, Red = significant in favour of (active) control group

^a^Analysis based only on data from the hospital with an active control group

**Fig. 4:**
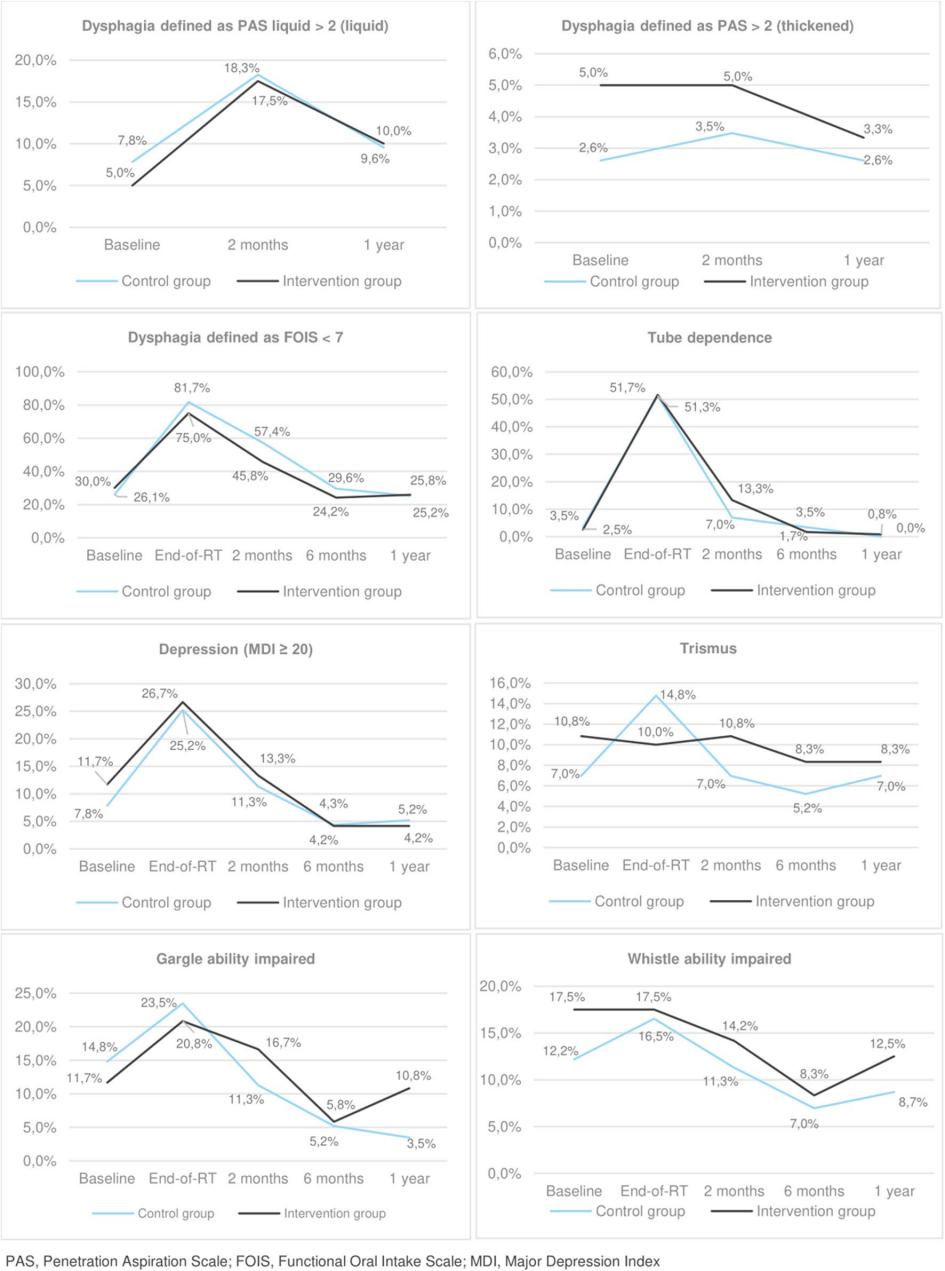
Symptoms and side effects over time

At baseline 15 (6%) participants presented with decreased swallow safety for liquid (PAS > 2), 21 (9%) had trismus, 66 (28%) was limited in their oral diet (FOIS ≤ 6), 7 (3%) dependent on tube feeding, 31 (13%) had impaired gargle function, 35 (15%) could not whistle and 23 (10%) had symptoms of depression (MDI ≥ 20). Figure 4 provides an overview of the development of impairment for both groups at all follow-ups. An elaborate table can be found in online resource 3. Raw data graphs on all outcomes and in four groups; intervention groups and control groups at each hospital, respectively, are provided in online resource 4.

There were no adverse events related to the swallowing intervention, and only few adverse events during or immediately after PRT: dyspnoea (*n* = 2), flare-up of old injury (*n* = 2), nausea and vomiting (*n* = 2), nerve- and muscle pain (*n* = 1), dizziness (*n* = 1), joint pain (*n* = 1).

Adherence to swallowing intervention was analysed on a sub-sample (*n* = 45) in another study [24]. Participants were prescribed an average of 6.5 unique exercises [range 4–14], and median adherence to self-administered exercise was 78%. Median attendance to supervised sessions was 100% (range 53–100%) [24].

Fourteen participants provided comments in the questionnaires related to their satisfaction with the trial. Comments were divided into (1) improved motivation, (2) better coping with treatment, (3) a sense of limited late effects, (4) gratefulness for social and emotional support pointing to the benefits of the intervention outside the a priori decided outcomes.

## Discussion

This randomized study demonstrates the importance of comparing a swallowing exercise intervention to a non-active control group. While the trial cannot support the hypothesis that swallowing exercises performed during radiotherapy improves penetration and aspiration scores for HNC patients, some significant outcomes can be observed on mouth opening, depression, anxiety, pain on NRS and EORTC H&N35, insomnia and use of pain killers at 1 year after end-of-treatment when analysing data from the hospital with a non-active control group only as was done in post hoc analyses.

Both groups, intervention and control, share the same pattern of an initial decline in functional level during or immediately after treatment with a subsequent increase until 1-year follow-up. HNC treatment is intensive causing substantial decline on many functional levels [35]. Our study reflects that such decline is inevitable despite a simultaneous exercise regime. While our intervention included what we assumed to be essential elements of a successful intervention including frequent supervision with easy access, logbooks to support home-based exercise, and individualized programs to support meaningfulness and usefulness, we still did not find consistent effects. Several explanations are plausible. First, when initiating the trial, it looked to be the first RCT of its kind with a non-active control group. Soon after, however, HNC radiotherapy was decentralized and so to reach full power, we included patients from an additional hospital which had implemented swallowing counselling to all HNC patients referred to radiotherapy. Secondly, in the 3.5 years recruitment period we observed an increasing awareness on dysphagia. Health care staff in hospitals and municipality-based rehabilitation centres increasingly advocated the importance of actively maintaining swallowing function to patients, who also had improved access to information. In 2019 the Copenhagen Municipality and the Danish Ministry of Health launched two public HNC rehabilitation sites aimed at healthcare professionals and patients, respectively [36, 37]. Unfortunately, we were not able to collect data on non-trial related rehabilitation services or therapeutic interventions received that could impact the difference in outcome between groups.

Thirdly, if swallowing impairment is due to an underlying fibrotic condition as a result of radiotherapy a continued progression should be expected, and maintenance of an exercise program will be crucial to maintain function over time [38]. As the tested intervention closed at end-of-treatment the—perhaps—long-term effects of the exercises remain to be seen. While we found high adherence to the swallowing intervention [24] poor adherence in HNC patients is a known obstacle in exercise trials [20]. Although participants were encouraged to continue with swallowing exercises after end-of-treatment, this was not a protocolized intervention. Preventive swallowing studies commonly test a program consisting of the same exercises to all participants. While we assumed that swallowing intervention needs to differ substantially between patients, we deliberately chose to make room for individual needs in the composition of each exercise program. This makes it difficult to compare our results with other studies, and to describe the intervention carried out in general terms. However, we find, that this personalized approach is more relevant in a clinical setting characterized by a variation in the nature and severity of side effects between patients. Ultimately the number and type of exercises may not matter as much as the frequency of which they are performed, and to what extend the patient can maintain an oral diet. Hutcheson et al. [10] support this in a retrospective observational study of 497 pharyngeal cancer patients, and found that maintaining an oral diet throughout radiotherapy was a strong predictor for long-term functional swallowing.

Possibly, our intervention would have benefited from a stronger focus on how to maintain an oral diet alongside the swallowing exercises. Nutritional counselling was usual care, although primarily focussing on maintaining an adequate nutritional level and preventing weight loss [39]. Maintenance of a nutritionally adequate oral diet will likely best be obtained in the collaboration between a dietitian and a speech-language-pathologist/OT [40].

Interestingly, Messing et al. [15] found similar negative results in another RCT with no difference between groups for neither functional swallowing outcomes, QoL, tube dependence, nor PAS among 60 patients with pharyngeal, laryngeal or glottic cancer, randomized to either Therabite only or Therabite and swallow exercises. However, the authors did report significant differences for some physiological swallowing outcomes with better swallow efficiency and less pharyngeal phase impairments in the intervention group [15].

A priori, we assumed that a bimodal intervention would have a better overall effect on patients’ physical, mental and social functioning. Although we expect that maintenance of LBM will affect the general functional level and overall health, the physical exercise modality may have been too unspecific in a trial primarily expecting improved swallowing outcomes, and too short in duration at a critical point in patients’ treatment trajectory to expect significant effect on physical outcomes. Importantly, however, the intervention did not harm any participants, and generally the satisfaction with the intervention was positive among participants. Participant feedback indicates that the intervention provides a certain ‘feel-good’ sense in HNC patients, who exercise during treatment.

Notably, in post hoc analyses carried out on each hospital separately it appeared that when comparing an intervention group with a non-active control group we were able to find significant differences between groups in favour of intervention group on several outcome measures including fatigue, depression and swallowing. For seven different outcomes we found differences at 1 year after treatment, compared to no differences in the initial analyses including both hospitals. Further, the differences between groups were generally, to a larger extent than in the full sample analyses, clinically relevant. For instance, we found a significant and clinically meaningful difference in fatigue between groups, and a difference in patient reported swallowing at 6 months after treatment. Sleep was the most consistent outcome where the intervention group suffered from insomnia significantly less than the control group at end-of-treatment and 6 and 12 months after treatment. While there were also some differences between groups at the hospital with an active control group, they were few, less frequently clinically relevant and mainly found at end-of-treatment. These results may indicate that the intervention does have a positive effect after all, also on a longer term.

## Conclusion

This randomized controlled trial on preventive swallowing exercises and progressive resistance training during radiotherapy did not show an effect on swallowing safety in HNC patients measured by penetration and aspiration. Possible explanations relate to the intervention duration being too short, lacking an integration of exercises into survivorship, and to the real difference between groups being too small. However, comparing intervention to a non-active control group only, significant effects were found on mouth opening, HRQOL, depression and anxiety 1 year after end of treatment. Despite lacking evidence of the effect of preventive swallowing exercises, HNC patients suffer from substantial late effects related to swallowing and radiation-induced fibrosis that compromises the range of motion, strength and coordination of swallowing structures that can occur years after treatment. The need to identify long-lasting intervention to mitigate these functional deteriorations is ever more crucial as the surviving HNC population is increasing and comparing intervention to non-active controls is crucial for the interpretation of results on future swallowing studies.

## Supplementary Information

Below is the link to the electronic supplementary material.

Supplementary file1 (PDF 234 kb)

Supplementary file2 (PDF 168 kb)

Supplementary file3 (PDF 188 kb)

Supplementary file4 (PDF 1206 kb)

## References

[1] Hutcheson KA, Lewin JS (2012). Functional outcomes after chemoradiotherapy of laryngeal and pharyngeal cancers. Curr Oncol Rep.

[2] Bressan V, Stevanin S, Bianchi M, Aleo G, Bagnasco A, Sasso L (2016). The effects of swallowing disorders, dysgeusia, oral mucositis and xerostomia on nutritional status, oral intake and weight loss in head and neck cancer patients: a systematic review. Cancer Treat Rev.

[3] Mortensen HR, Overgaard J, Jensen K (2013). Factors associated with acute and late dysphagia in the DAHANCA 6 & 7 randomized trial with accelerated radiotherapy for head and neck cancer. Acta Oncol.

[4] Langendijk JA, Doornaert P, Verdonck-de Leeuw IM, Leemans CR, Aaronson NK, Slotman BJ (2008). Impact of late treatment-related toxicity on quality of life among patients with head and neck cancer treated with radiotherapy. J Clin Oncol.

[5] Daugaard R, Kjaer T, Johansen C, Christiansen J, Andersen E, Nielsen AL, Dalton SO (2017). Association between late effects assessed by physicians and quality of life reported by head-and-neck cancer survivors. Acta Oncol.

[6] Ward EC, van As-Brooks CJ (2014). Head and neck cancer. Treatment, rehabilitation, and outcomes.

[7] Mittal BB, Pauloski BR, Haraf DJ, Pelzer HJ, Argiris A, Vokes EE, Rademaker A, Logemann JA (2003). Swallowing dysfunction—preventative and rehabilitation strategies in patients with head-and-neck cancers treated with surgery, radiotherapy, and chemotherapy: a critical review. Int J Radiat Oncol Biol Phys.

[8] Murphy BA, Gilbert J (2009). Dysphagia in head and neck cancer patients treated with radiation: assessment, sequelae, and rehabilitation. Semin Radiat Oncol.

[9] Carnaby-Mann G, Crary MA, Schmalfuss I, Amdur R (2012). “Pharyngocise”: randomized controlled trial of preventative exercises to maintain muscle structure and swallowing function during head-and-neck chemoradiotherapy. Int J Radiat Oncol Biol Phys.

[10] Hutcheson KA, Bhayani MK, Beadle BM, Gold KA, Shinn EH, Lai SY, Lewin J (2013). Eat and exercise during radiotherapy or chemoradiotherapy for pharyngeal cancers: use it or lose it. JAMA Otolaryngol Head Neck Surg.

[11] Kotz T, Federman AD, Kao J, Milman L, Packer S, Lopez-Prieto C, Forsythe K, Genden EM (2012). Prophylactic swallowing exercises in patients with head and neck cancer undergoing chemoradiation: a randomized trial. Arch Otolaryngol Head Neck Surg.

[12] van der Molen L, van Rossum MA, Rasch CR, Smeele LE, Hilgers FJ (2014). Two-year results of a prospective preventive swallowing rehabilitation trial in patients treated with chemoradiation for advanced head and neck cancer. Eur Arch Otorhinolaryngol.

[13] Mortensen HR, Jensen K, Aksglaede K, Lambertsen K, Eriksen E, Grau C (2015). Prophylactic swallowing exercises in head and neck cancer radiotherapy. Dysphagia.

[14] Van Den Berg MGA, Kalf JG, Hendriks JCM, Takes RP, Van Herpen CML, Wanten GJA, Drenth JPH, Kaanders JHAM, Merkx MAW (2016). Normalcy of food intake in patients with head and neck cancer supported by combined dietary counseling and swallowing therapy: a randomized clinical trial. Head Neck.

[15] Messing BP, Ward EC, Lazarus CL (2017). Prophylactic swallow therapy for patients with head and neck cancer undergoing chemoradiotherapy: a randomized trial. Dysphagia.

[16] Kulbersh BD, Rosenthal EL, McGrew BM, Duncan RD, McColloch NL, Carroll WR, Magnuson JS (2006). Pretreatment, preoperative swallowing exercises may improve dysphagia quality of life. Laryngoscope.

[17] Carroll WR, Locher JL, Canon CL, Bohannon IA, McColloch NL, Magnuson JS (2008). Pretreatment swallowing exercises improve swallow function after chemoradiation. Laryngoscope.

[18] Duarte VM, Chhetri DK, Liu YF, Erman AA, Wang M (2013). Swallow preservation exercises during chemoradiation therapy maintains swallow function. Otolaryngol Head Neck Surg.

[19] Virani A, Kunduk M, Fink DS, McWhorter AJ (2015). Effects of 2 different swallowing exercise regimens during organ-preservation therapies for head and neck cancers on swallowing function. Head Neck.

[20] Perry A, Lee SH, Cotton S, Kennedy C (2016). Therapeutic exercises for affecting post-treatment swallowing in people treated for advanced-stage head and neck cancers. Cochrane Database Syst Rev.

[21] Silver HJ, Dietrich MS, Murphy BA (2007). Changes in body mass, energy balance, physical function, and inflammatory state in patients with locally advanced head and neck cancer treated with concurrent chemoradiation after low-dose induction chemotherapy. Head Neck.

[22] Lonbro S, Dalgas U, Primdahl H, Johansen J, Nielsen JL, Aagaard P, Hermann AP, Overgaard J, Overgaard K (2013). Progressive resistance training rebuilds lean body mass in head and neck cancer patients after radiotherapy—results from the randomized DAHANCA 25B trial. Radiother Oncol.

[23] Hajdu SF, Wessel I, Johansen C, Kristensen CA, Kadkhoda ZT, Plaschke CC, Dalton SO (2017). Swallowing therapy and progressive resistance training in head and neck cancer patients undergoing radiotherapy treatment: randomized control trial protocol and preliminary data. Acta Oncol.

[24] Hajdu SF, Christensen MB, Kristensen MO, Wessel I, Johansen C, Dalton S (2019). Adherence to preventive swallowing exercises for head and neck cancer patients undergoing (chemo)radiotherapy treatment. Acta Oncol.

[25] The International Dysphagia Diet Standardisation Initiative 2016 (2019). Complete IDDSI Framework Detailed definitions 2.0. https://ftp.iddsi.org/Documents/Complete_IDDSI_Framework_Final_31July2019.pdf. Accessed 29 Apr 2020.

[26] Neubauer PD, Rademaker AW, Leder SB (2015). The Yale Pharyngeal Residue Severity Rating Scale: an anatomically defined and image-based tool. Dysphagia.

[27] Aaronson NK, Ahmedzai S, Bergman B (1993). The European Organization for Research and Treatment of Cancer QLQ-C30: a quality-of-life instrument for use in international clinical trials in oncology. J Natl Cancer Inst.

[28] Bjordal K, Hammerlid E, Ahlner-Elmqvist M (1999). Quality of life in head and neck cancer patients: validation of the European Organization for Research and Treatment of Cancer Quality of Life Questionnaire-H&N35. J Clin Oncol.

[29] Chen AY, Frankowski R, Bishop-Leone J, Hebert T, Leyk S, Lewin J, Goepfert H (2001). The development and validation of a dysphagia-specific quality-of-life questionnaire for patients with head and neck cancer. Arch Otolaryngol Head Neck Surg.

[30] Hutcheson KA, Lewin JS, Barringer DA, Lisec A, Gunn GB, Moore MW, Holsinger FC (2012). Late dysphagia after radiotherapy-based treatment of head and neck cancer. Cancer.

[31] Hutcheson KA, Barringer DA, Rosenthal DI, May AH, Roberts DB, Lewin JS (2008). Swallowing outcomes after radiotherapy for laryngeal carcinoma. Arch Otolaryngol Head Neck Surg.

[32] Jack S, West M, Grocott MP (2011). Perioperative exercise training in elderly subjects. Best Pract Res Clin Anaesthesiol.

[33] Speck RM, Courneya KS, Masse LC, Duval S, Schmitz KH (2010). An update of controlled physical activity trials in cancer survivors: a systematic review and meta-analysis. J Cancer Surviv.

[34] Mishra SI, Scherer RW, Snyder C, Geigle PM, Berlanstein DR, Topaloglu O (2012). Exercise interventions on health-related quality of life for people with cancer during active treatment. Cochrane Database Syst Rev.

[35] Murphy BA, Deng J (2015). Advances in supportive care for late effects of head and neck cancer. J Clin Oncol.

[36] Sundhed.dk (2020). Rehabilitering ved hoved-halskræft—Borger. https://www.sundhed.dk/borger/behandling-og-rettigheder/kraeft/hoved-halskraeft/. Accessed 26 May 2020.

[37] Sundhed.dk (2019). Rehabilitering ved hoved-halskræft—Sundhedsprofessionel.

[38] Stubblefield MD (2011). Radiation fibrosis syndrome: neuromuscular and musculoskeletal complications in cancer survivors. PM R.

[39] Sundhedsstyrelsen (2015). Opfølgningsprogram for Hoved- og halskræft.

[40] Kristensen MB, Isenring E, Brown B (2020). Nutrition and swallowing therapy strategies for patients with head and neck cancer. Nutrition.

