# Supplementary material for: Swallowing Exercise During Head and Neck Cancer Treatment: Results of a Randomized Trial
Source: Dysphagia. 2021 Jun 11;37(4):749–62. doi: 10.1007/s00455-021-10320-5 (PMC9345844; doi:10.1007/s00455-021-10320-5)
Supplement: Supplementary file 1 — Supplementary file1 (PDF 234 kb) [file 455_2021_10320_MOESM1_ESM.pdf]

Online resource 4: Swallowing intervention guide

Swallowing exercise sessions are planned and conducted three times weekly by occupational therapist (OT). Sessions should preferably be organized with one day in between sessions. If the patient receives chemotherapy treatment one weekly swallowing session should be conducted during chemo infusion at the oncology department.
All sessions are individual and should be scheduled right before or after radiotherapy treatment to reduce waiting time in the hospital for the patient as much as possible.
Before the first session the OT should be well oriented in the patient's medical journal, specifically information related to tumour location, comorbidities and any surgery prior to radiotherapy.

CLINICAL EVALUATION

Before tailoring a swallowing exercise program take the following into consideration:
Generally:

- Conversation about eating and drinking activities, including eating habits, weight and pain with a focus on what has/has not changed.
- Observe:
  - Breathing (natural, strained, superficial)
  - Posture
  - Facial expressions (general impression, symmetry, coordination of oral movement)
  - Voice/voice changes (hoarse, wet, thick, weak)
  - Swallow of saliva / drooling
  - Cough strength

Visual examination:

- Facial expression: Any asymmetry or coordination impairments is being related to muscle strength and sensibility
- Jaw, mouth, tongue:
  - Mouth opening and closing (range of motion, trismus, tension in mm.masseter)
  - Mucous membranes in mouth (colour and condition), sensibility in mouth
  - Tongue; sensibility, mobility, tonus, coordination, strength

Tactile examination:

- Tonus and sensibility in mouth and tongue (spontaneous swallow after stimuli, strength in tongue movements)

Screening of oral intake:
If found relevant or necessary, observe the intake of food or liquid.

SELECTING EXERCISES

Each swallowing exercise program should be individually tailored to each patient. Jaw mobility exercises to prevent trismus (exercises 7-9) is a minimum for all participants, except patients with small laryngeal cancers. Use the table below as a guideline when choosing exercises, keeping in mind they are prophylactic exercises so include radiation field and expected side effects to treatment in the overall evaluation.
Even though only few exercises are chosen, try to instruct the patient in all exercises from the beginning. Some exercises are difficult to learn, e.g. Mendelsohn, so it is important that the patient is familiar with the exercise in case it will be relevant to include it in the program later when side effects to treatment can complicate the performance of the exercise further.

Online resource 4: Swallowing intervention guide

Exercise	What does it do/train	When to use it	Typical tumour site[i]	N.B. / possible contra	Instruction
1. Reach tongue forward and back<br>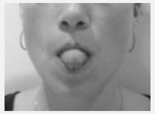	Selective movement of tongue & mobility of base of tongue	Reduced ability to process, control and manipulate bolus due to reduced tongue function	Tongue<br>Oral cavity		Stick your tongue out as far as possible, hold the stretch for 5 sec. Try to keep your tongue in the middle.<br><br>Relax.<br><br>Pull back your tongue as far as you can. Imagine you must scratch the back of your throat with the back of your tongue. Hold for 5 sec<br><br>Relax<br><br>Repeat as many times as you can, up to 10 times.
2. Tongue to cheek<br>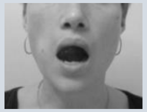	Selective movement of tongue	Reduced ability to process, control and manipulate bolus due to reduced tongue function	Tongue<br>Oral cavity		Place the tip of the tongue in your right cheek, as far back in the mouth as you can, hold the stretch<br><br>Relax<br><br>Repeat the exercise, this time to the left.<br><br>Relax<br><br>Repeat as many times as you can, up to 10 times.
3. Tongue to mouth corners<br>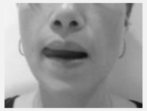	Selective movement of tongue	Reduced tongue coordination<br><br>Reduced ability to process, control and manipulate bolus due to reduced tongue function.	Tongue<br>Oral cavity		Smile. Place the tip of the tongue in the right corner of your mouth. Then move it the left mouth corner. Shift back and forth.<br><br>Practice moving the tongue faster from side to side, still being precise with the tip of the tongue to the mouth corners.<br>Repeat as many times as you can, up to 10 times.

[i] This is a guideline and not an exhaustive list. Which exercises to choose will largely depend on prior surgery, tooth extraction, side effects to treatment, treatment site, pain level etc.

Online resource 4: Swallowing intervention guide

**Exercise**	**What does it do/train**	**When to use it**	**Typical tumour site**[i]	**N.B. / possible contra**	**Instruction**
4. Resistance to tongue 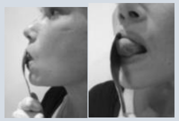	Isometric strength of tongue musculature	Reduced tongue mobility and strength<br><br>Reduced control and manipulation of bolus	Tongue<br>Oral cavity<br>Base of tongue	Open wound on the tongue	Stick your tongue out as far as you can and press a spoon against the tip of your tongue. Hold for 5 sec.<br><br>Relax.<br><br>Stick your tongue out as far as you can and press a spoon against the side of your tongue. Hold for 5 sec.<br><br>Relax.<br><br>Repeat on the opposite side.<br><br>Repeat as many times as you can, up to 5 times in all 3 directions.
5. Gargle 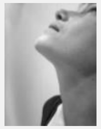	Mobility of base of tongue	Reduced mobility at base of tongue<br><br>Patients who describe tightness or a different sensation when yawning, e.g. after tongue base resection	Base of tongue	Aspiration	Look to the ceiling.<br><br>Gargle for 3 sec.<br><br>Relax.<br><br>Repeat as many times as you can, up to 10 times.<br><br>You can do the exercise with or without water in your mouth. Notice what feels okay for you.
6. Yawn	Mobility of base of tongue	Reduced mobility at base of tongue<br><br>Patients who describe tightness or different sensation when yawning, e.g. after tongue base resection	Base of tongue<br>Tonsils	Can provoke gag reflex	Open your mouth wide.<br><br>Start yawning, like when you are tired. You will feel like all the muscles in your throat open up.<br><br>Relax.<br><br>Repeat as many times as you can, up to 10 times.

[i] This is a guideline and not an exhaustive list. Which exercises to choose will largely depend on prior surgery, tooth extraction, side effects to treatment, treatment site, pain level etc.

Online resource 4: Swallowing intervention guide

Exercise	What does it do/train	When to use it	Typical tumour site[i]	N.B. / possible contra	Instruction
7. Mouth opening 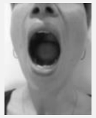	Jaw mobility	Reduced mouth opening / jaw mobility<br><br>Reduced chewing function	Tongue<br>Oral cavity<br>Oropharynx<br>UPT		Open your mouth wide as much as you can.<br>Keep the stretch for 10 sec.<br><br>Relax for 5-10 sec.<br><br>Repeat as many times as you can, up to 10 times.
8. Jaw; side to side 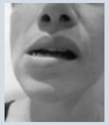	Jaw mobility	Reduced mouth opening / jaw mobility<br><br>Reduced chewing function	Tongue<br>Jaw<br>Oropharynx<br>UPT		Move the mandible slowly from side to side.<br><br>Hold the stretch while counting to 5 in each side.<br><br>Repeat as many times as you can, up to 10 times to each side.
9. Jaw; undershot 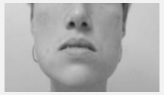	Jaw mobility	Reduced mouth opening / jaw mobility<br>Reduced chewing function	Tongue<br>Jaw<br>Oropharynx<br>UPT		Shoot the mandible forward to make undershot.<br><br>Hold the stretch for 5 sec<br><br>Relax<br><br>Repeat up to 10 times
10. Valsalva 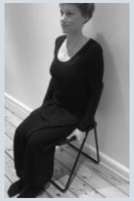	Closing mechanism to the airway and oppression of air in the throat (requires complete movement of vocal folds)	Patients who aspirate or have difficulties closing off the airway<br><br>Reduced mobility of vocal folds	Vocal folds	Surgery in the past 14 days<br><br>Heart conditions<br><br>Uncontrolled hypertension	Take a deep breath.<br><br>Hold your breath.<br><br>Do not try to hold your breath with your lips. Hold your breath in the throat like, for instance, when you lift something heavy.<br><br>Hold for 5 sec.<br><br><u>If it is difficult:</u><br>Sit on a chair<br>Place both hands under the chair and pull upwards<br>Keep ”lifting” while holding your breath for 5 sec

---

[i] This is a guideline and not an exhaustive list. Which exercises to choose will largely depend on prior surgery, tooth extraction, side effects to treatment, treatment site, pain level etc.

Online resource 4: Swallowing intervention guide

Exercise	What does it do/train	When to use it	Typical tumour site[i]	N.B. / possible contra	Instruction
11. Head lift<br>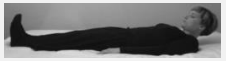	Muscles of the hyoid bone for an improved opening to the oesophagus.	Pharyngeal residue<br><br>Aspiration<br><br>Reduced laryngeal mobility<br><br>Poor opening of the upper oesophageal sphincter	Jaw<br>Hypopharynx<br>Larynx	Neck problems<br><br>Exhaustion / overtraining	Lie flat on your back, preferably without a pillow under your head.<br><br>Lift your head from the ground and look towards your feet.<br><br>Hold this position as long as you can, up to 1 minute. Make sure you only lift your head using the muscles in the neck. Shoulders should stay in the ground.<br><br>Lie the head back and relax for 1 minute.<br><br>Repeat twice more.<br><br>Then:<br>Lift your head up once again before placing it back in the ground in one smooth movement.<br><br>Repeat this movement as many times as you can, up to 30 times.
12. Mendelsohn<br>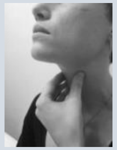	Prolongs duration of swallow<br><br>Facilitates laryngeal elevation<br><br>Improves coordination of swallow	Pharyngeal residue<br><br>Aspiration<br><br>Reduced laryngeal mobility/elevation<br><br>Reduced coronation of breath and swallow	Jaw<br>Hypopharynx<br>Larynx	Shortness of breath<br><br>Trouble with breath holding<br><br>Difficult exercise to learn	First, swallow normally. Feel with your fingers on your neck how the larynx moves up and down.<br><br>On your next swallow, feel the larynx move up and hold it their using the muscles of your throat.<br><br>Try not to lift your larynx too early. Let it lift naturally and hold it for three sec. Use the muscles in your throat to keep it lifted, not your fingers.<br><br>Relax and complete the swallow<br><br>Repeat as many times as you can, up to 10 times.

[i] This is a guideline and not an exhaustive list. Which exercises to choose will largely depend on prior surgery, tooth extraction, side effects to treatment, treatment site, pain level etc.

Online resource 4: Swallowing intervention guide

Exercise	What does it do/train	When to use it	Typical tumour site[i]	N.B. / possible contra	Instruction
13. Masako 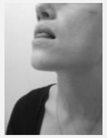	Improved contact between base of tongue and pharyngeal wall	Reduced mobility of tongue or base of tongue<br>Weak base of tongue<br><br>Reduced mobility of posterior pharyngeal wall<br><br>Pharyngeal residue	Tongue<br>Base of tongue	Coughing<br><br>Worsening of symptoms	Place the tip of the tongue between your front teeth (without biting your tongue).<br><br>Swallow while you keep the tongue in place between your teeth.<br><br>Repeat as many times as you can, up to 10 times.
14. Effortful swallow	Improved oral and pharyngeal pressure on bolus	Reduced strength in tongue and pharynx, including reduced pharyngeal contraction<br><br>Ineffective or slow swallow<br><br>Pharyngeal residual	Tongue<br>Hard palate<br>Soft palate<br>Tonsil<br>Hypopharynx	Difficult to monitor	Imagine you must swallow something large. Swallow with extra effort.<br><br>The extra effort should be visible on your neck when you swallow<br><br>Repeat as many times as you can, up to 10 times.
15. (super) Supra-glottic swallow	Facilitates closure of airway/airway protection<br><br>Facilitates improved coordination of swallow and breath	Poor closure to the airway<br><br>Reduced coordination of breathing and swallowing<br><br>Reduced laryngeal mobility		Shortness of breath<br><br>Trouble with breath holding	Following is a technique you can apply when you eat or drink. This should improve the coordination of swallow and breath. If you have a tendency to aspirate your food or drinks, this technique can help you protect your lungs before the food or liquid reaches the upper airway.<br><br>The first time you try this technique you should do it only with saliva. Then you can try to do it with a bit of liquid. When you feel familiar with the technique you can apply it when you eat and/or drink.<br><br>1. Take a deep breath and hold it<br>2. Take a bite or sip<br>3. Swallow while still holding your breath until all food or liquid is gone from your mouth<br>4. Cough right after you swallow (on the exhale)<br>5. Swallow again<br>6. Breathe<br>Repeat step 1-6 at with each swallow.

[i] This is a guideline and not an exhaustive list. Which exercises to choose will largely depend on prior surgery, tooth extraction, side effects to treatment, treatment site, pain level etc.

Online resource 4: Swallowing intervention guide

Article title: Swallowing exercises during head and neck cancer treatment – results of a randomized trial

Journal: *Dysphagia*.

Authors: Hajdú SF, Wessel I, Dalton SO, Eskildsen, SJ, Johansen C.

Corresponding author: Sara Fredslund Hajdú, dept of occupational therapy and physiotherapy, Copenhagen University Hospital Rigshospitalet, Denmark & Cancer Late Effects Research Unit (CASTLE), Department of Oncology, Copenhagen University Hospital Rigshospitalet, Denmark. sara.fredslund.hajdu@regionh.dk
