# Supplementary material for: Swallowing Exercise During Head and Neck Cancer Treatment: Results of a Randomized Trial
Source: Dysphagia. 2021 Jun 11;37(4):749–62. doi: 10.1007/s00455-021-10320-5 (PMC9345844; doi:10.1007/s00455-021-10320-5)
Supplement: Supplementary file 2 — Supplementary file2 (PDF 168 kb) [file 455_2021_10320_MOESM2_ESM.pdf]

Online resource 2: Effect of intervention at end-of-treatment and 2, 6 and 12 months after end-of-treatment: Results of analyses of association between group allocation (intervention group vs. control group) and healthy functional level (binary outcomes) of 235 HNC patients, SYNK trial, 2015-2019, with adjustment for HPV(p16), sex, age group, marital status, tumor-site and chemo.

	**No. of cases with normal function (%)**				
	Intervention group n=120	Control group n=115	OR	95% CI	*p*
**PAS, liquid consistency, with scores < 3**					
2 months	76 (63)	76 (66)	0.88	[0.38; 2.03]	0.77
1 year	78 (65)	75 (65)	0.61	[0.24; 1.57]	0.31
**PAS, honey consistency, with scores < 3**					
2 months	91 (76)	93 (81)	1.10	[0.33; 3.62]	0.88
1 year	86 (72)	83 (72)	0.98	[0.24; 4.03]	0.98
**ECOG performance status with scores < 2**					
End of treatment	71 (59)	73 (63)	1.09	[0.55; 2.15]	0.80
2 months	97 (81)	94 (82)	1.20	[0.39; 3.64]	0.75
6 months	83 (69)	77 (67)	0.93	[0.14; 6.10]	0.94
12 months	92 (77)	88 (77)	0.37	[0.03; 5.01]	0.45
**MID ≥ 35mm**					
End of treatment	83 (69)	84 (73)	2.03	[1.11; 3.69]	**0.02**
2 months	90 (75)	90 (78)	0.74	[0.39; 1.41]	0.36
6 months	71 (59)	70 (61)	0.43	[0.19; 0.96]	**0.04**
12 months	82 (68)	80 (70)	0.49	[0.21; 1.16]	0.11
**FOIS with scores = 7 (no restrictions)**					
End of treatment	8 (7)	11 (10)	0.85	[0.33; 2.23]	0.75
2 months	48 (40)	35 (30)	1.61	[0.88; 2.92]	0.12
6 months	55 (46)	46 (37)	1.30	[0.70; 2.43]	0.41
12 months	62 (52)	61 (53)	0.79	[0.43; 1.46]	0.45
**Gargle (yes)**					
End of treatment	70 (58)	69 (60)	0.81	[0.37; 1.78]	0.61
2 months	80 (67)	82 (71)	0.51	[0.25; 1.20]	0.13
6 months	72 (60)	69 (60)	0.78	[0.26; 2.27]	0.64
12 months	74 (62)	78 (68)	0.16	[0.04; 0.65]	**0.01**
**Whistle (yes)**					
End of treatment	74 (62)	79 (69)	0.89	[0.40; 1.98]	0.78
2 months	87 (73)	84 (73)	0.83	[0.37; 1.90]	0.67
6 months	73 (61)	69 (60)	0.78	[0.30; 2.04]	0.61
12 months	75 (63)	77 (67)	0.54	[0.22; 1.29]	0.17

HNC, Head & Neck Cancer; OR, Odds Ratio; HPV, Human Papilloma Virus; CI, Confidence Interval; PAS, Penetration Aspiration Scale; ECOG, Eastern Cooperative Oncology Group; MID, Maximum Interincisal Distance; FOIS, Functional Oral Intake Scale

Article title: Swallowing exercises during head and neck cancer treatment – results of a randomized trial
Journal: *Dysphagia*.
Authors: Hajdú SF, Wessel I, Dalton SO, Eskildsen, SJ, Johansen C.
Corresponding author: Sara Fredslund Hajdú, dept of occupational therapy and physiotherapy, Copenhagen University Hospital Rigshospitalet, Denmark & Cancer Late Effects Research Unit (CASTLE), Department of Oncology, Copenhagen University Hospital Rigshospitalet, Denmark.
sara.fredslund.hajdu@regionh.dk
