# Supplementary material for: Swallowing Exercise During Head and Neck Cancer Treatment: Results of a Randomized Trial
Source: Dysphagia. 2021 Jun 11;37(4):749–62. doi: 10.1007/s00455-021-10320-5 (PMC9345844; doi:10.1007/s00455-021-10320-5)
Supplement: Supplementary file 3 — Supplementary file3 (PDF 188 kb) [file 455_2021_10320_MOESM3_ESM.pdf]

Online resource 3: Participants presenting with and developing selected symptoms during the first year after end-of-treatment. Analysis of 235 head and neck cancer patients, SYNK trial, 2015-2018.

	Intervention group (n=120)					Control group (n=115)				
	Baseline<br>n (%)	T1<br>n (%)	T2<br>n (%)	T3<br>n (%)	T4<br>n (%)	Baseline<br>n (%)	T1<br>n (%)	T2<br>n (%)	T3<br>n (%)	T4<br>n (%)
Penetration/Aspiration liquid consistency										
Abnormal (PAS >2)	6 (5)		21 (18)		12 (10)	9 (8)		21 (18)		11 (10)
Normal PAS	113 (94)		76 (63)		78 (65)	106 (92)		76 (66)		75 (65)
Missing	1 (1)		23 (19)		30 (25)	-		18 (16)		29 (25)
Penetration/Aspiration honey consistency										
Abnormal (PAS >2)	6 (5)		6 (5)		4 (3)	3 (3)		4 (3)		3 (3)
Normal PAS	112 (93)		91 (76)		86 (72)	111 (96)		93 (81)		83 (72)
Missing	2 (2)		23 (19)		30 (25)	1 (1)		18 (16)		29 (25)
Mouth opening										
Trismus (MID <35)	13 (11)	12 (10)	13 (11)	10 (8)	10 (8)	8 (7)	17 (15)	8 (7)	6 (5)	8 (7)
MID ≥ 35mm	107 (89)	83 (69)	90 (75)	71 (59)	82 (68)	105 (91)	84 (73)	90 (78)	70 (61)	80 (70)
Missing	0	25 (21)	17 (14)	39 (33)	28 (23)	2 (2)	14 (12)	17 (15)	39 (34)	27 (23)
Functional Oral Intake										
Impaired (FOIS <7)	36 (30)	90 (75)	55 (46)	29 (24)	31 (26)	30 (26)	94 (82)	66 (57)	34 (30)	29 (25)
Normal	84 (70)	8 (7)	48 (40)	55 (46)	62 (52)	85 (74)	11 (10)	35 (30)	46 (40)	61 (53)
Missing	0	22 (18)	17 (14)	36 (30)	27 (23)	0	10 (9)	14 (12)	35 (30)	25 (22)
Tube Dependence										
Tube dependent	3 (3)	62 (52)	16 (13)	2 (2)	1 (1)	4 (3)	59 (51)	8 (7)	4 (3)	-
Complete oral diet	117 (98)	36 (30)	87 (73)	82 (86)	92 (77)	111 (97)	46 (40)	93 (81)	76 (66)	90 (78)
Missing	0	22 (18)	17 (14)	36 (30)	27 (23)	0	10 (9)	14 (12)	35 (30)	25 (22)
Gargle water										
Cannot gargle	14 (12)	25 (21)	20 (17)	7 (6)	13 (11)	17 (15)	27 (23)	13 (11)	6 (5)	4 (3)
Can gargle	102 (85)	70 (58)	80 (67)	72 (60)	74 (62)	88 (77)	69 (60)	82 (71)	69 (60)	78 (68)
Missing	4 (3)	25 (21)	20 (17)	41 (34)	33 (28)	10 (9)	19 (17)	20 (17)	40 (35)	33 (29)
Whistle with sound										
Cannot whistle	21 (18)	21 (18)	17 (14)	10 (8)	15 (13)	14 (12)	19 (17)	13 (11)	8 (7)	10 (9)
Can whistle	99 (83)	74 (62)	87 (73)	73 (61)	75 (63)	99 (86)	79 (69)	84 (73)	69 (60)	77 (67)
Missing	0	25 (21)	16 (13)	37 (31)	30 (25)	2 (2)	17 (15)	18 (16)	38 (33)	28 (24)
Depression, MDI										
Any level (≥ 20)	14 (12)	32 (27)	16 (13)	5 (4)	5 (4)	9 (8)	29 (25)	13 (11)	5 (4)	6 (5)
Mild (20-24)	6 (5)	14 (12)	4 (3)	1 (1)	2 (2)	3 (3)	11 (10)	2 (2)	4 ((3)	2 (2)

Online resource 3 continued: Participants presenting with and developing selected symptoms during the first year after end-of-treatment. Analysis of 235 head and neck cancer patients, SYNK trial, 2015-2018.

Moderate (25-29)	5 (4)	10 (8)	7 (6)	2 (2)	2 (2)	2 (2)	8 (7)	7 (6)	0	0
Severe (≥ 30)	3 (3)	8 (7)	5 (4)	2 (2)	1 (1)	4 (3)	10 (9)	4 (3)	1 (1)	4 (3)
None (<20)	102 (85)	52 (43)	81 (68)	78 (65)	79 (66)	100 (87)	50 (43)	78 (68)	68 (59)	78 (68)
Missing	4 (3)	36 (30)	23 (19)	37 (31)	36 (30)	6 (5)	36 (31)	24 (21)	42 (37)	31 (30)
BMI[8]										
Underweight (<18.5)	5 (4)	3 (3)	7 (6)	4 (3)	5 (4)	2 (2)	5 (4)	7 (6)	7 (6)	5 (4)
Normal (18.5-25)	44 (37)	43 (36)	50 (42)	42 (35)	40 (33)	44 (38)	48 (42)	53 (46)	42 (37)	38 (33)
Obese (>25)	68 (57)	42 (35)	43 (36)	37 (31)	46 (38)	69 (60)	44 (38)	41 (36)	30 (26)	47 (41)
Missing	3 (3)	32 (27)	20 (17)	37 (31)	29 (24)	0	18 (16)	14 (12)	36 (31)	25 (22)

T1-T4: End-of-treatment, and 2-, 6- and 12 months after treatment; MID: Maximal Interincical Distance; FOIS: Functional Oral Intake Scale; PAS: Penetration Aspiration Scale; MDI: Major Depression Inventory; BMI: Body Mass Index.

Article title: Swallowing exercises during head and neck cancer treatment – results of a randomized trial
Journal: *Dysphagia*.
Authors: Hajdú SF, Wessel I, Dalton SO, Eskildsen, SJ, Johansen C.
Corresponding author: Sara Fredslund Hajdú, dept of occupational therapy and physiotherapy, Copenhagen University Hospital Rigshospitalet, Denmark & Cancer Late Effects Research Unit (CASTLE), Department of Oncology, Copenhagen University Hospital Rigshospitalet, Denmark.
sara.fredslund.hajdu@regionh.dk
