# Supplementary material for: Swallowing Exercise During Head and Neck Cancer Treatment: Results of a Randomized Trial
Source: Dysphagia. 2021 Jun 11;37(4):749–62. doi: 10.1007/s00455-021-10320-5 (PMC9345844; doi:10.1007/s00455-021-10320-5)
Supplement: Supplementary file 4 — Supplementary file4 (PDF 1206 kb) [file 455_2021_10320_MOESM4_ESM.pdf]

Online resource 4: Raw data graphs on four group

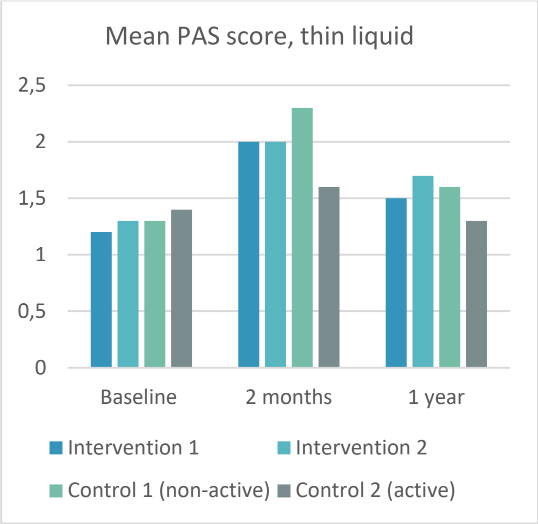

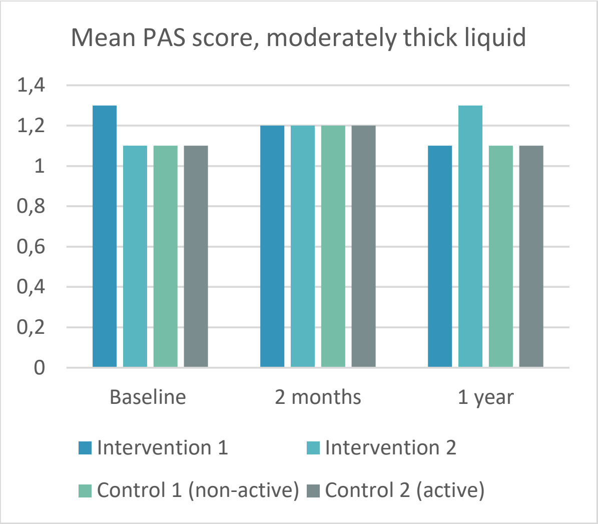

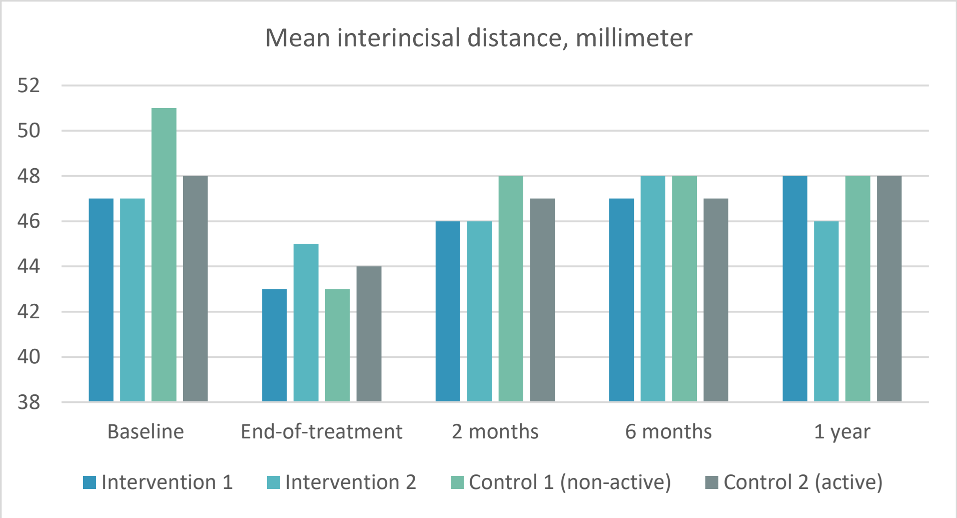

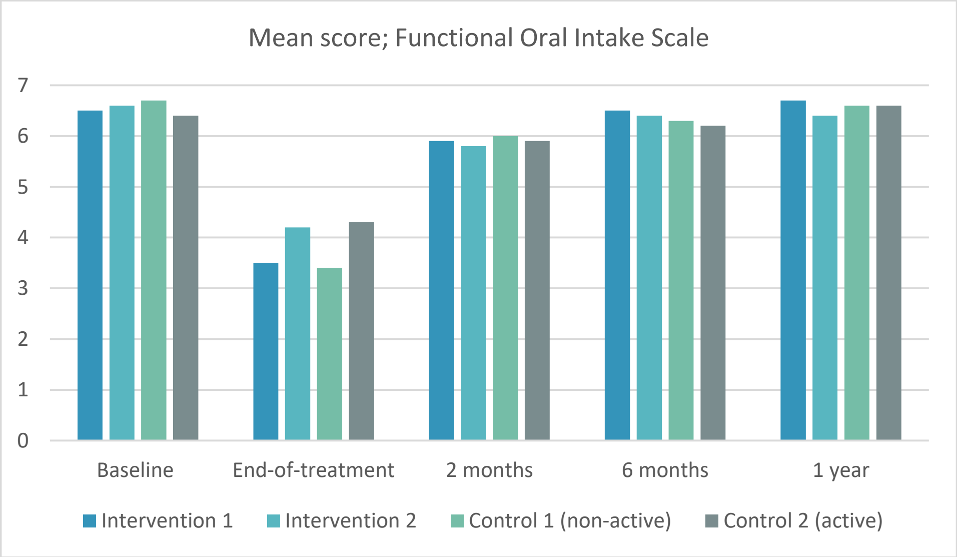

Online resource 4: Raw data graphs on four group

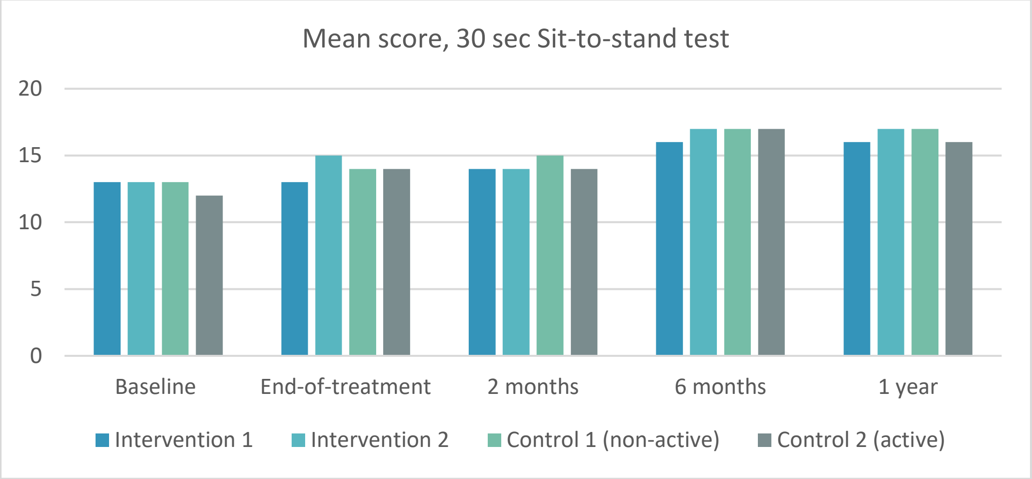

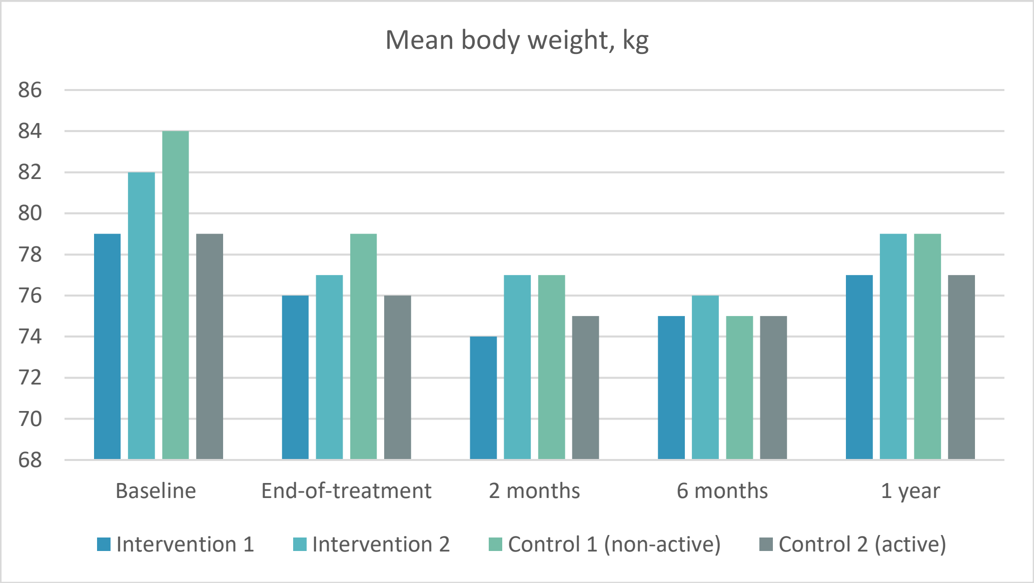

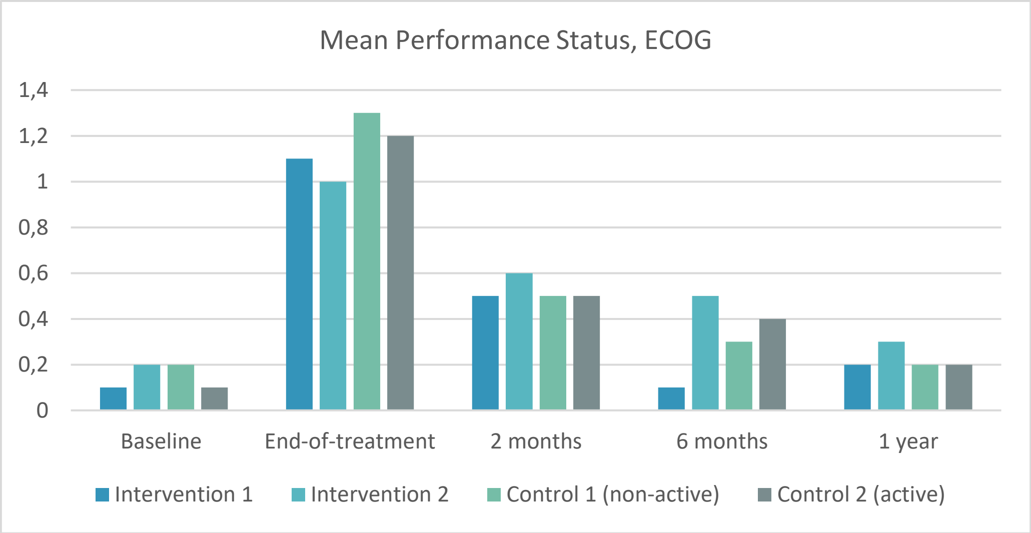

Online resource 4: Raw data graphs on four group

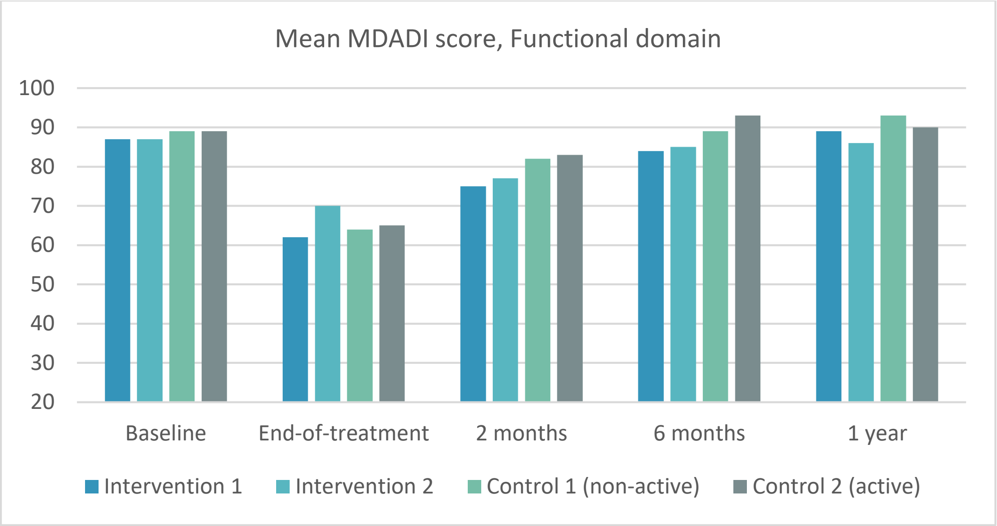

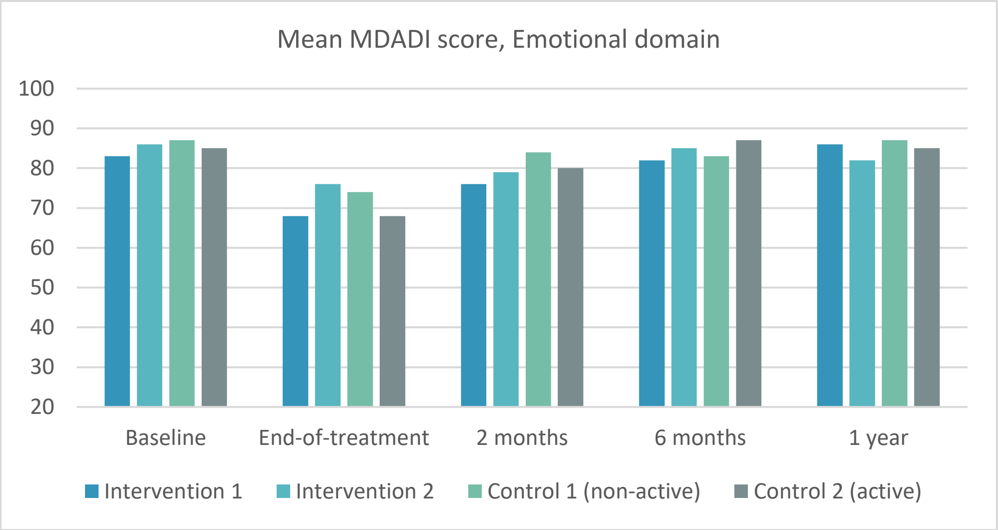

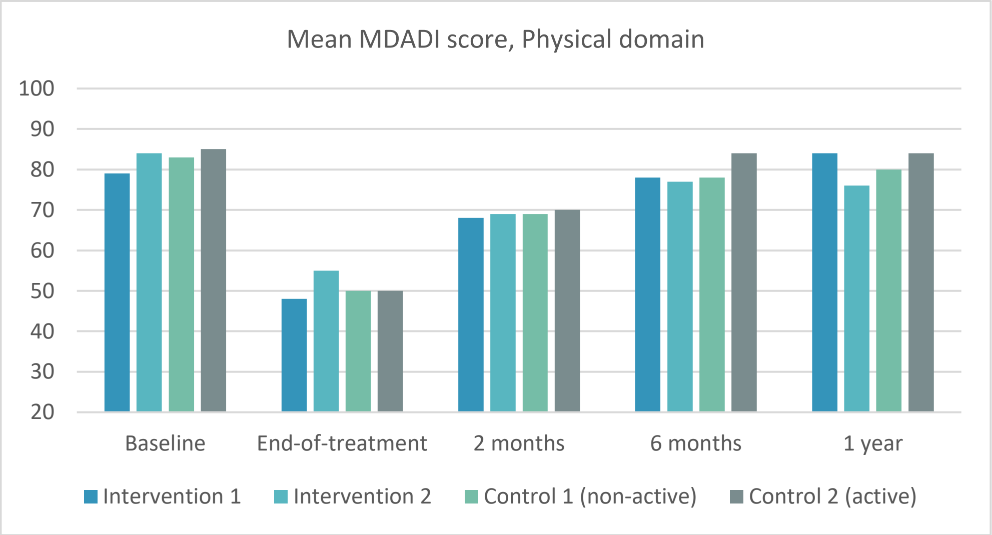

Online resource 4: Raw data graphs on four group

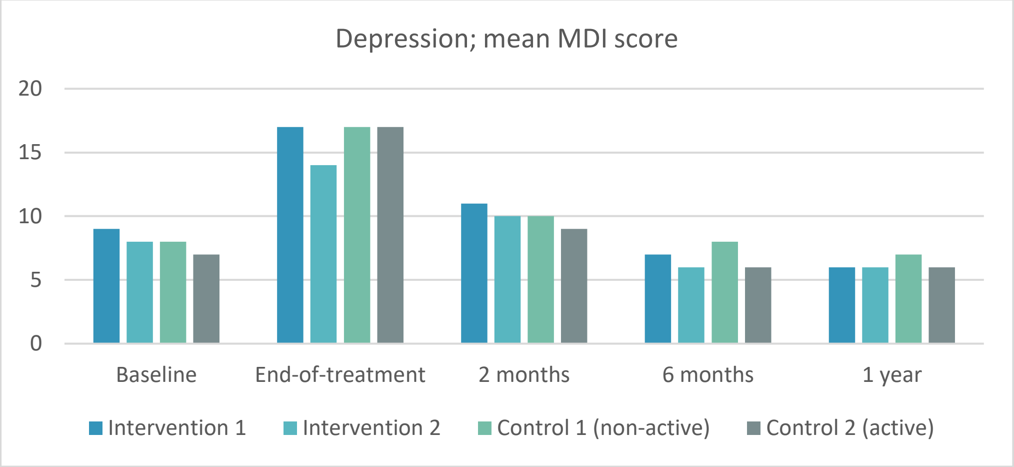

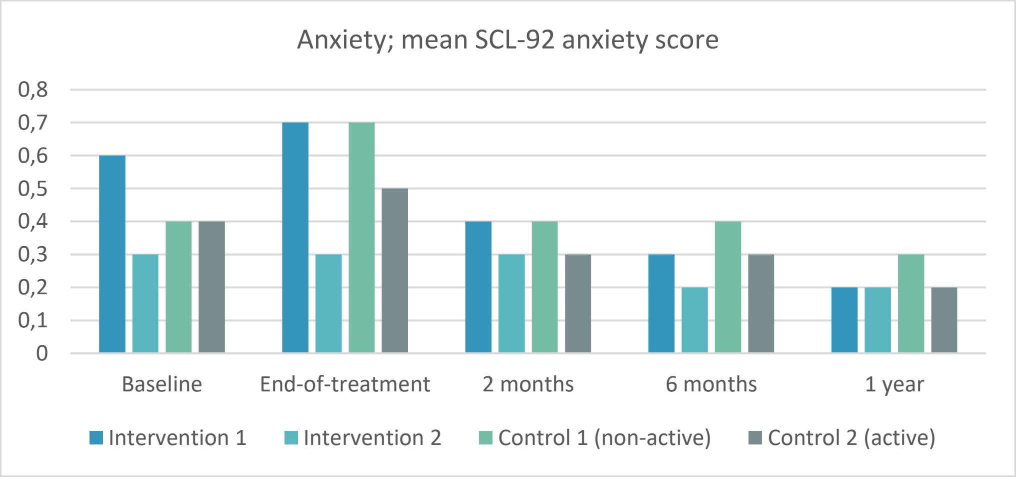

Online resource 4: Raw data graphs on four group

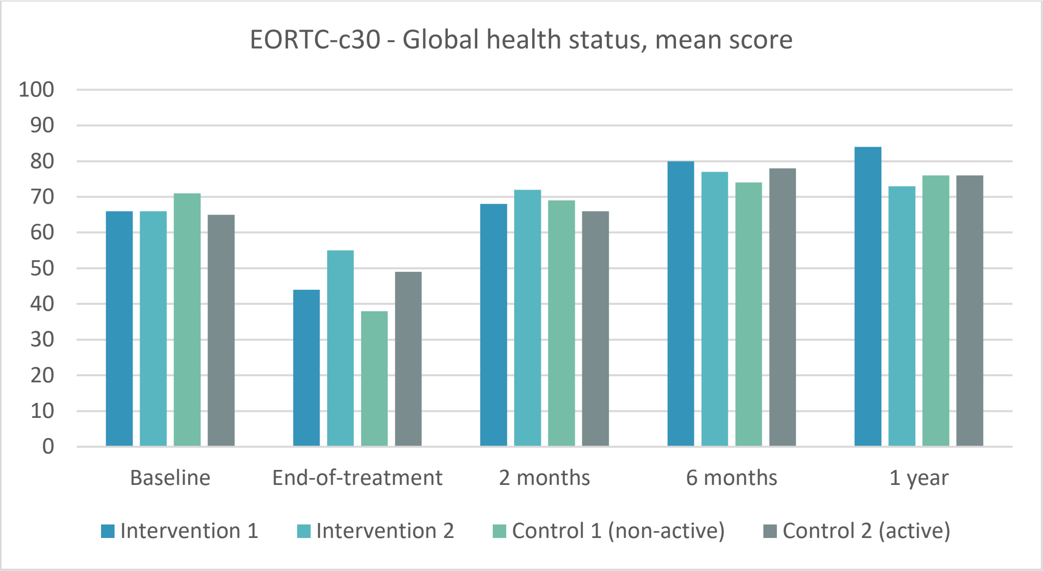

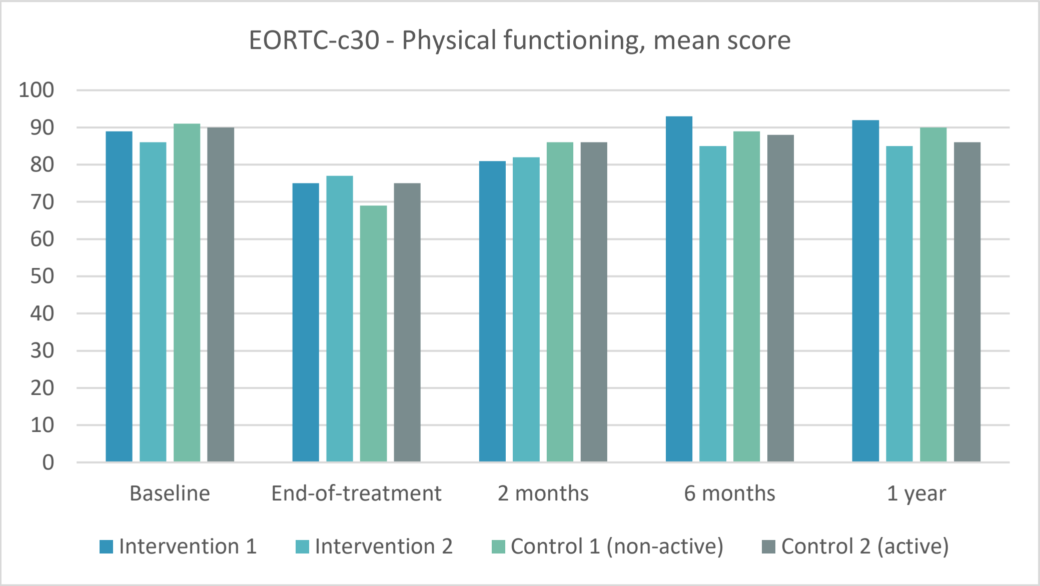

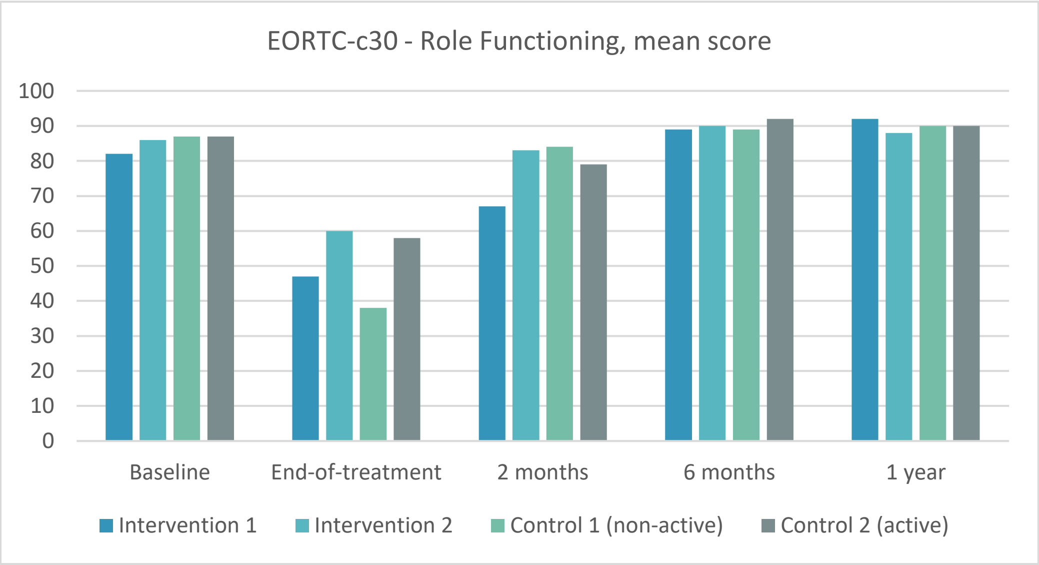

Online resource 4: Raw data graphs on four group

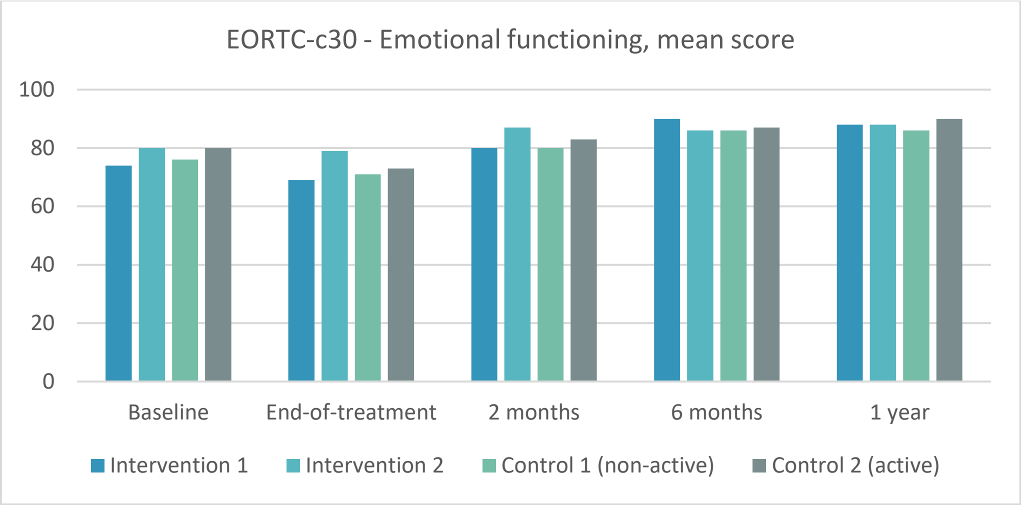

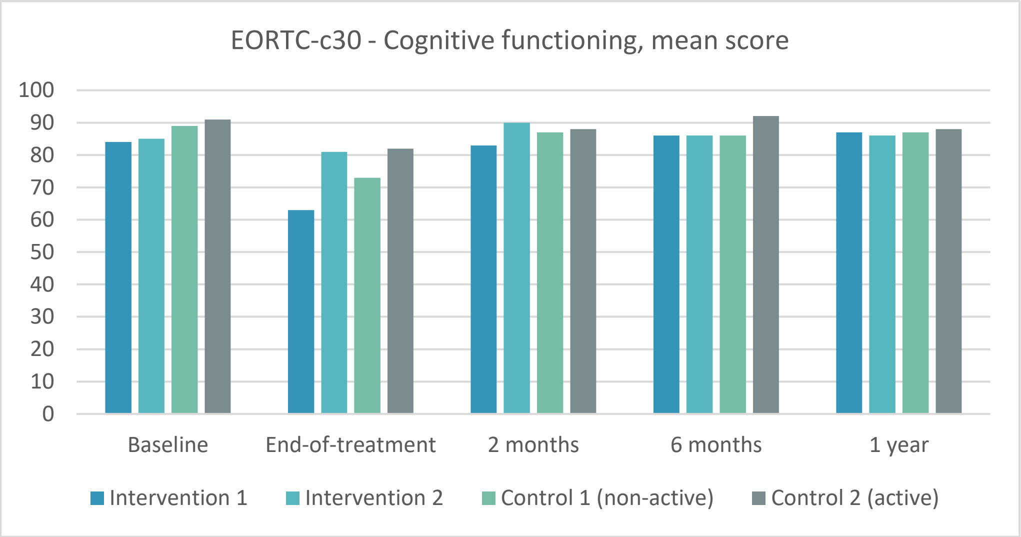

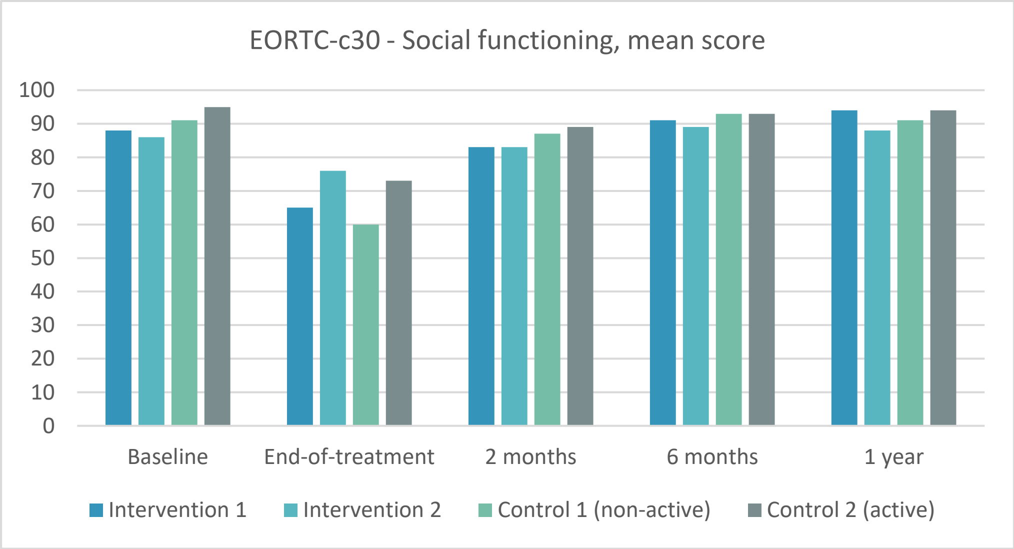

Online resource 4: Raw data graphs on four group

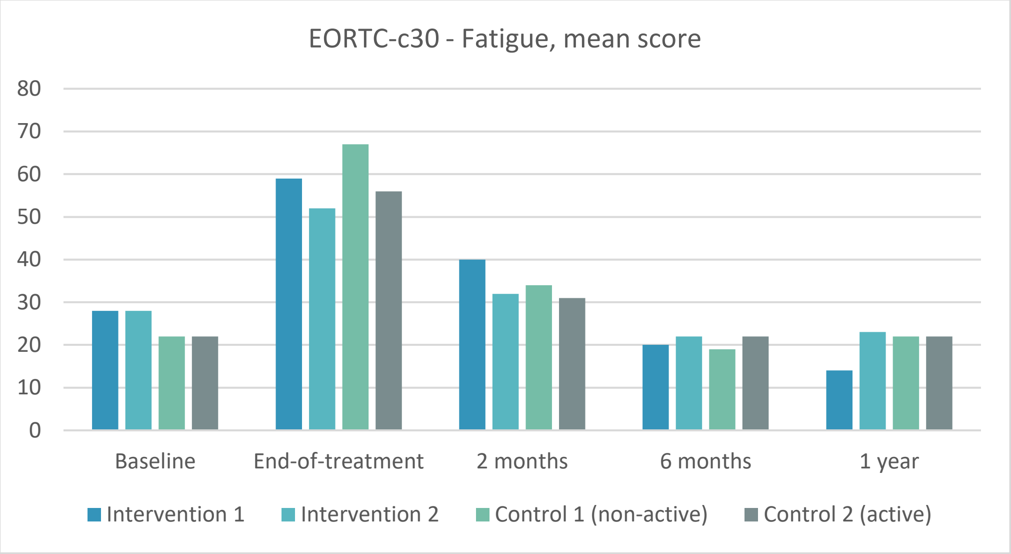

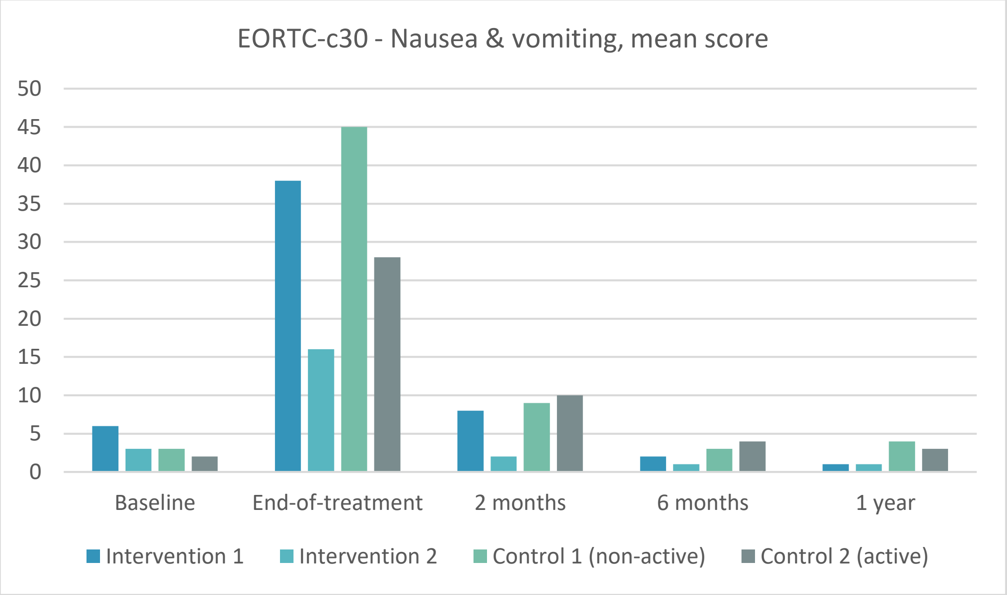

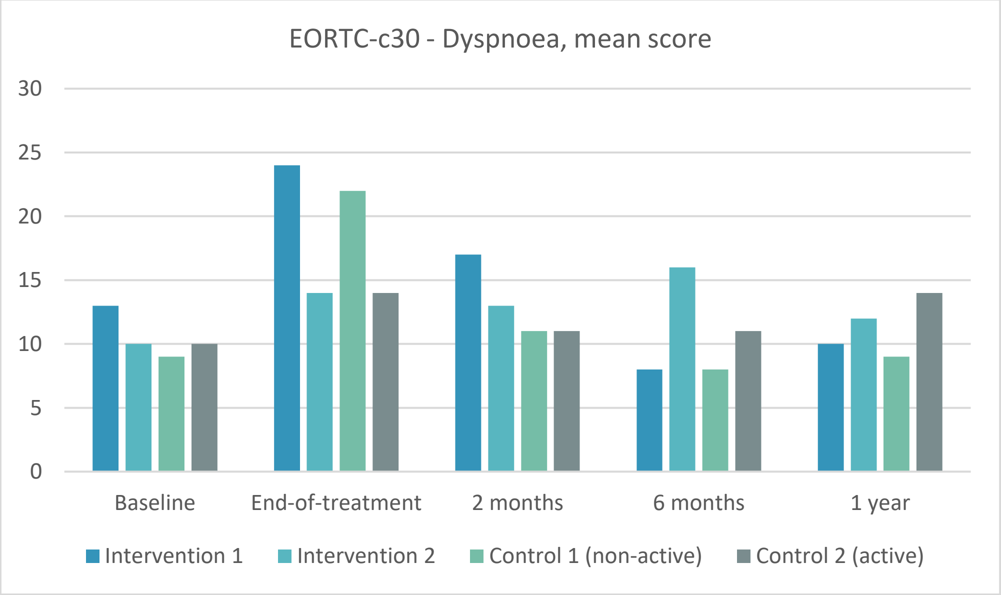

Online resource 4: Raw data graphs on four group

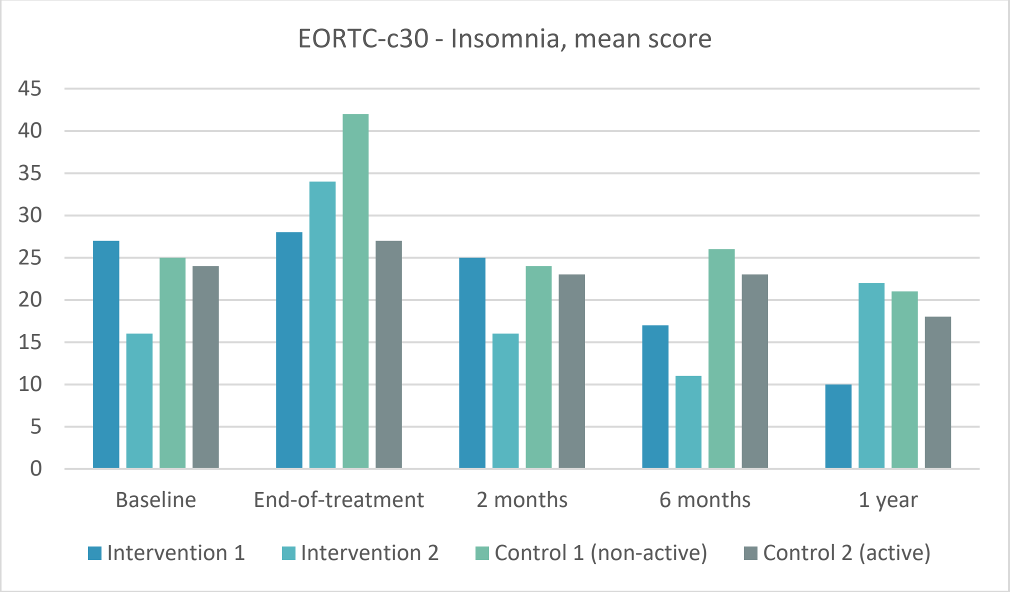

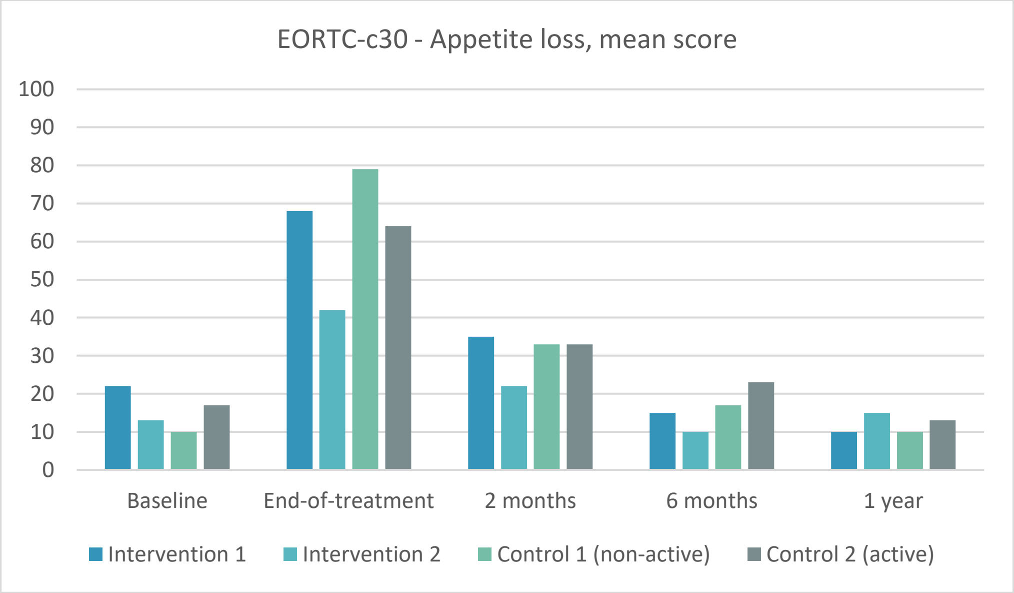

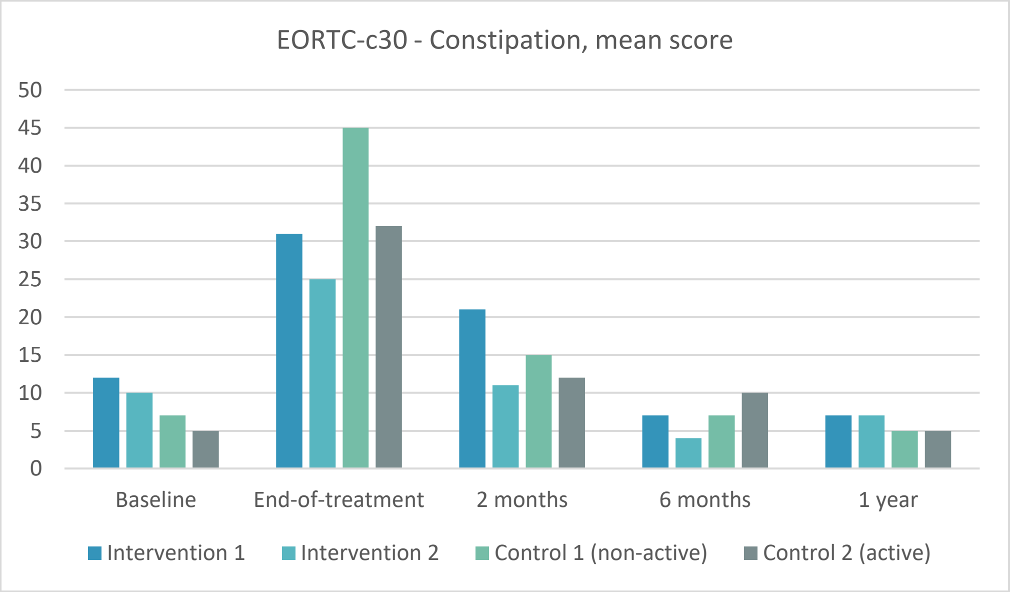

Online resource 4: Raw data graphs on four group

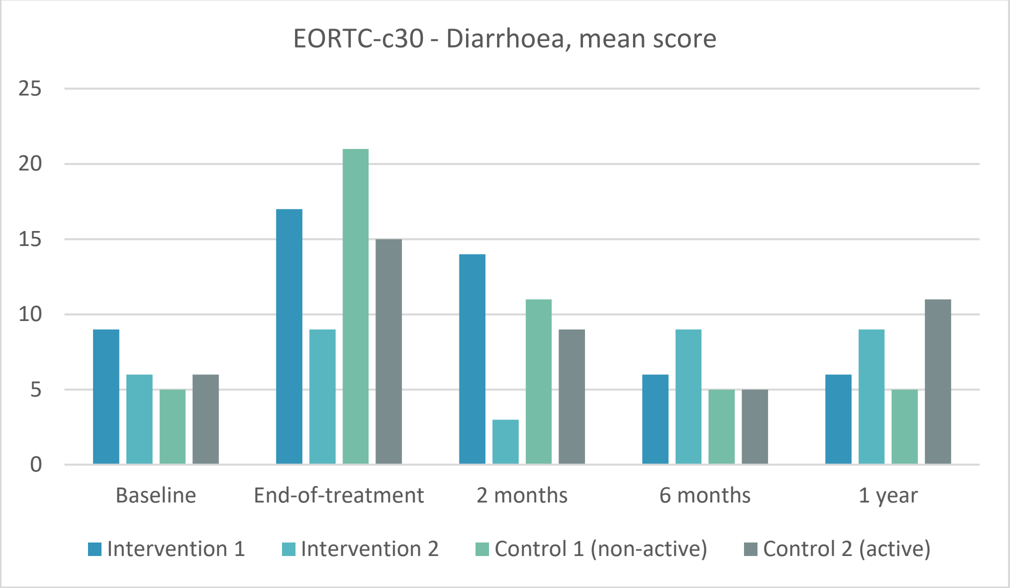

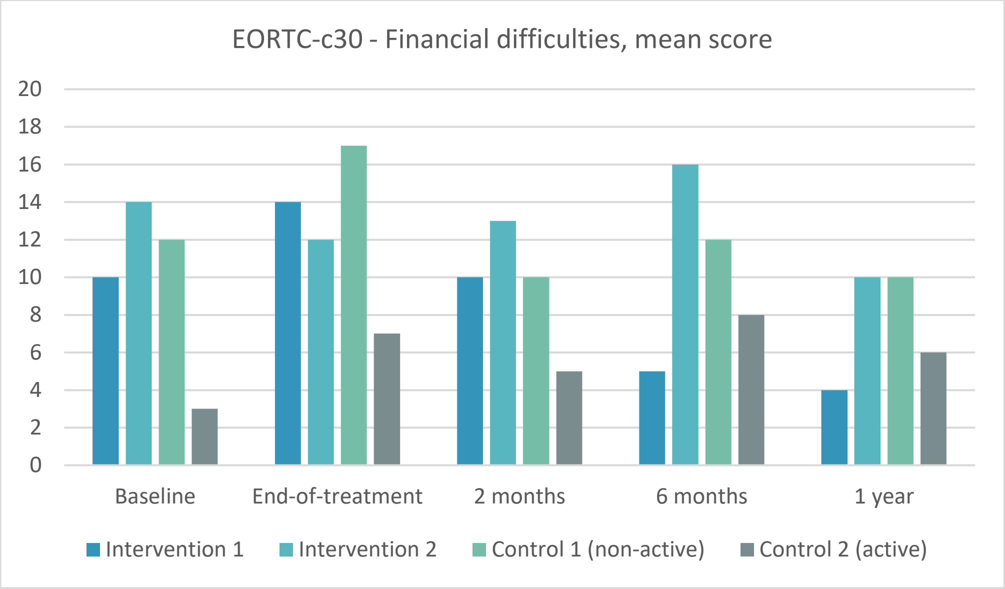

Online resource 4: Raw data graphs on four group

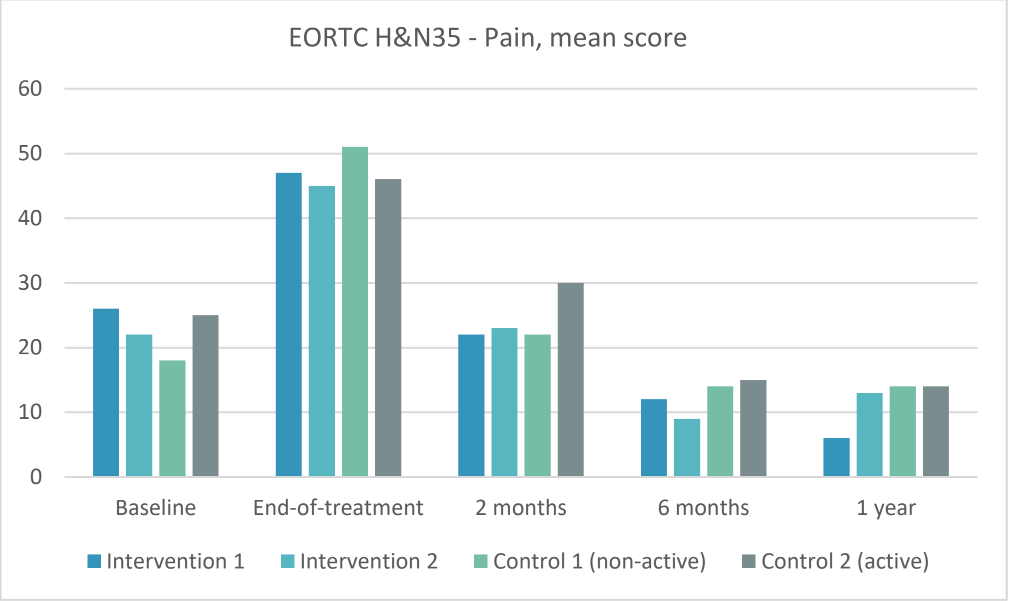

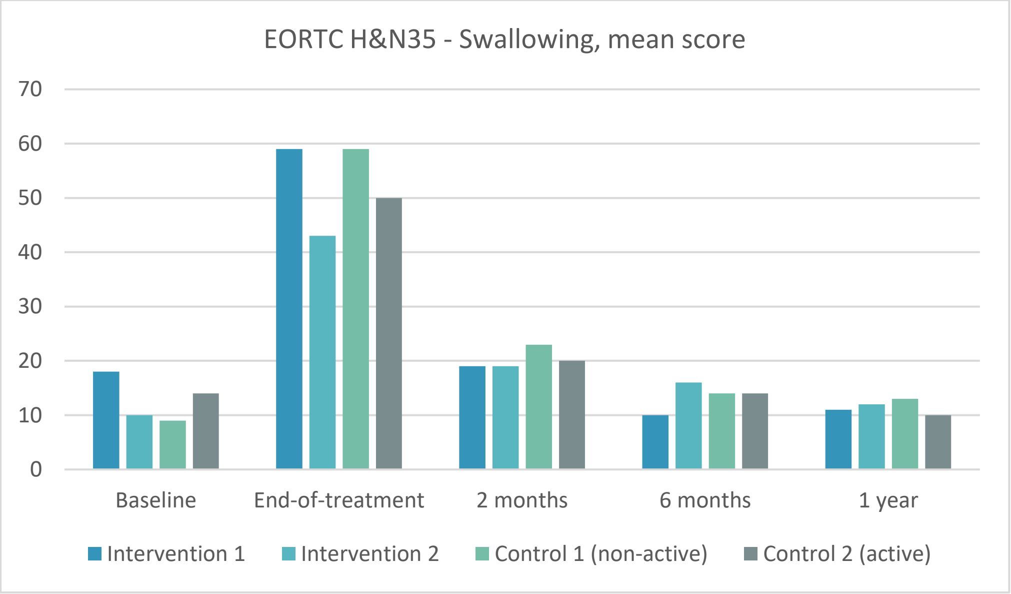

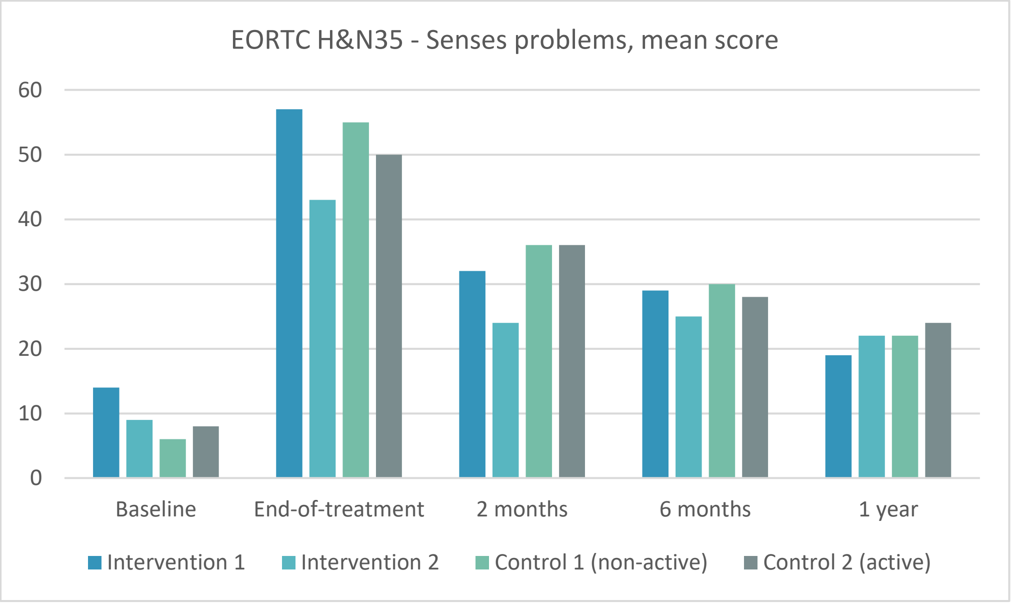

Online resource 4: Raw data graphs on four group

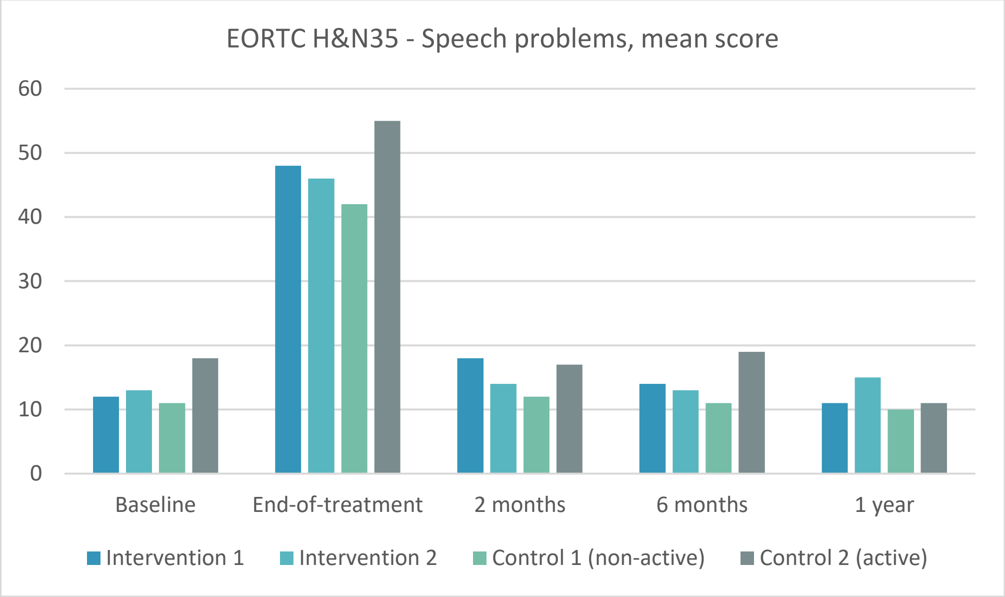

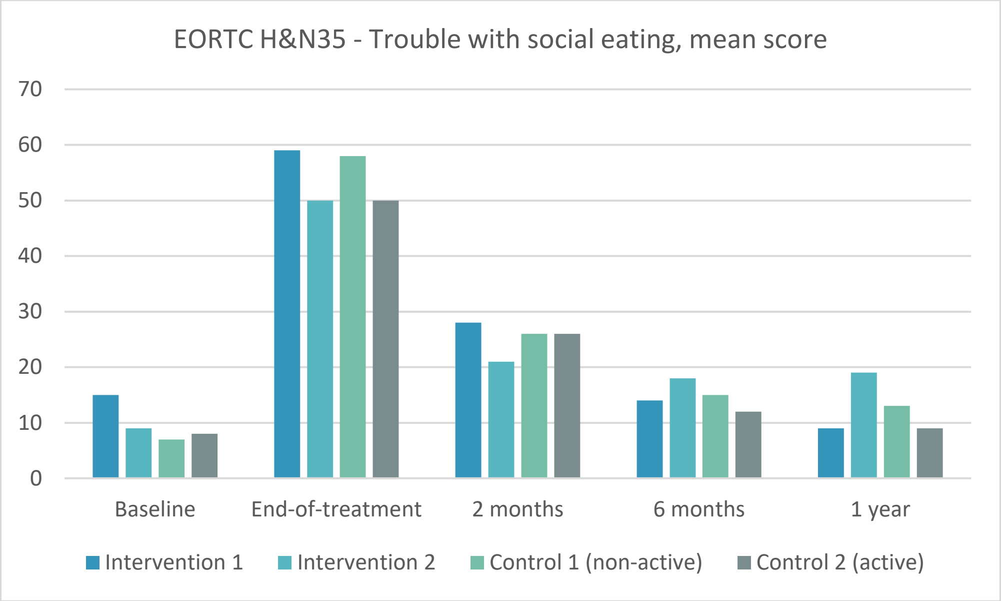

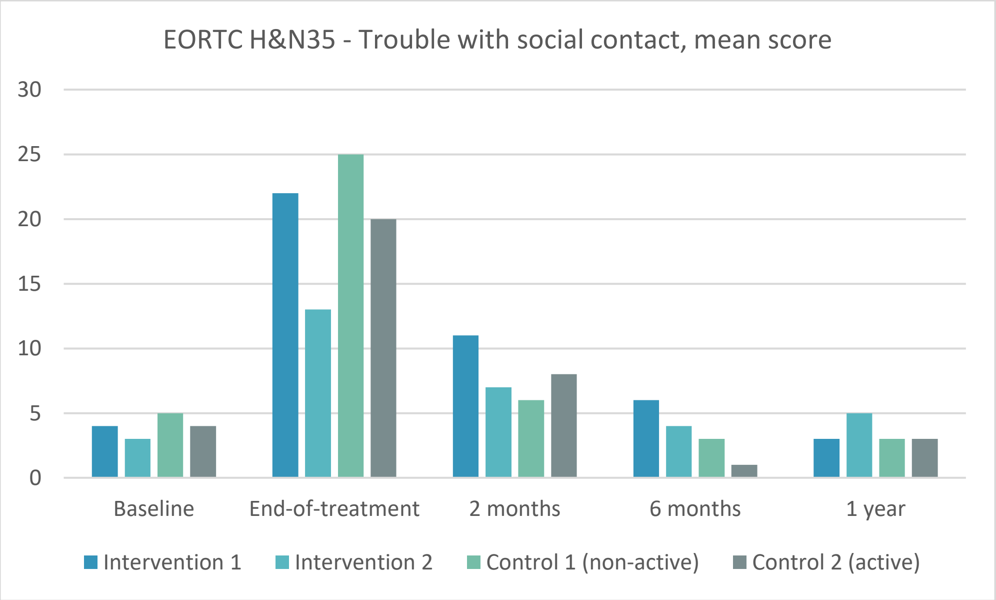

Online resource 4: Raw data graphs on four group

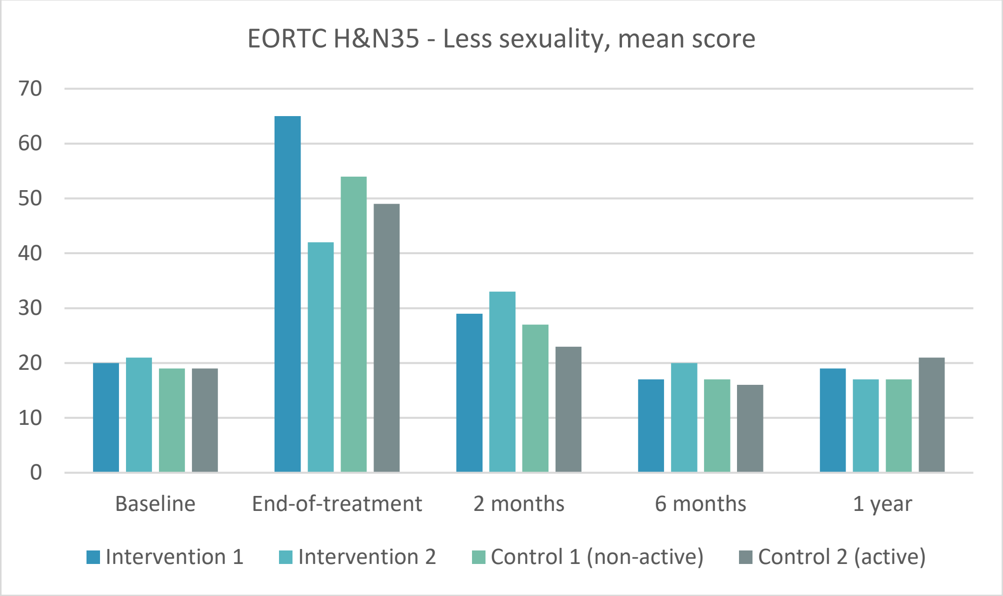

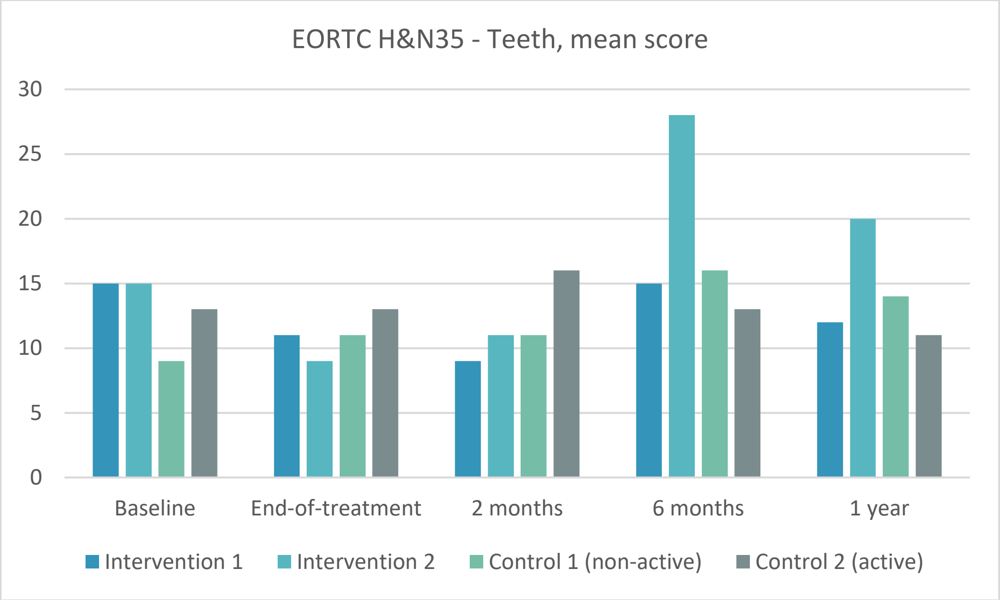

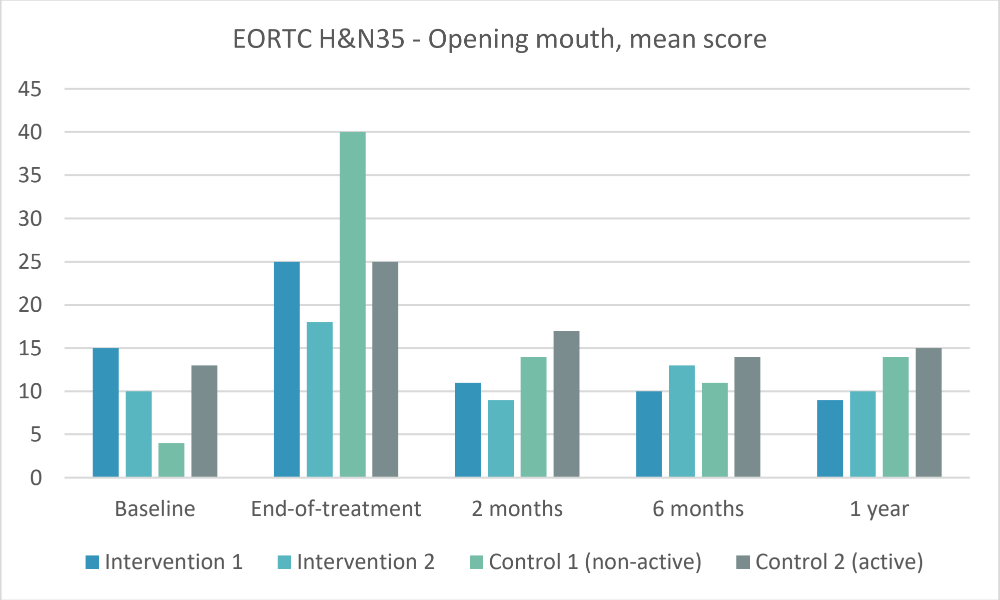

Online resource 4: Raw data graphs on four group

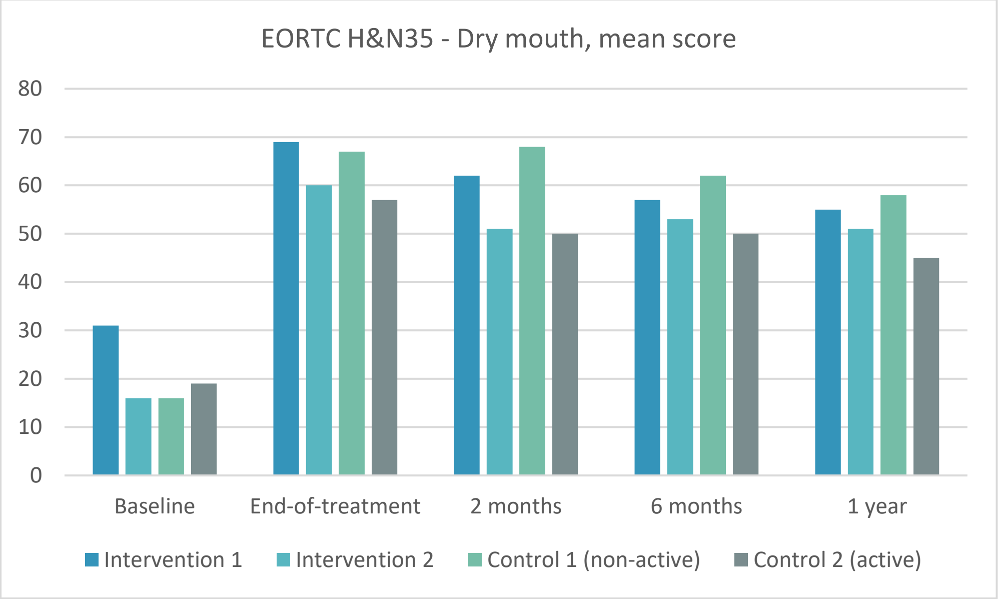

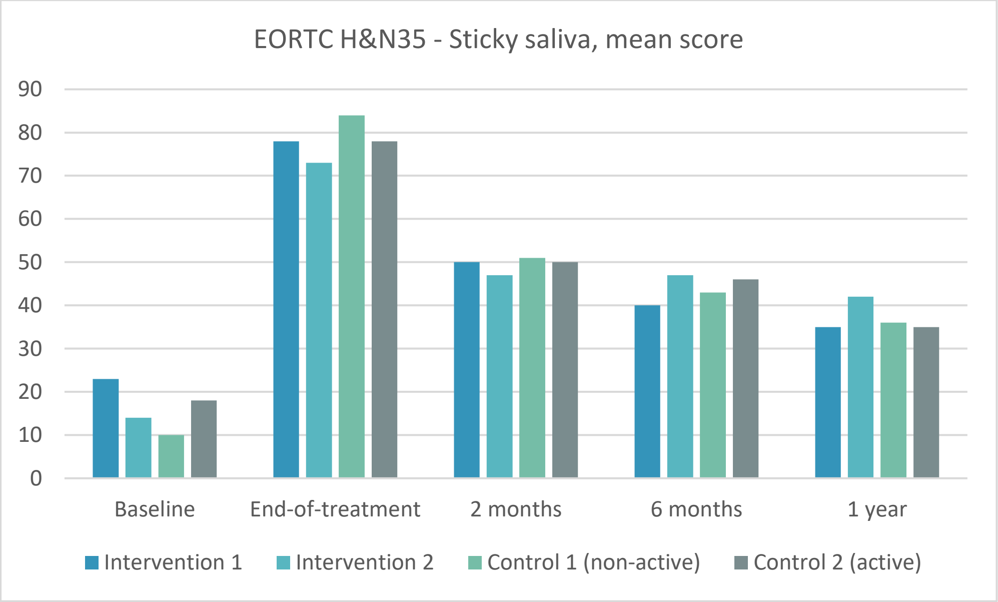

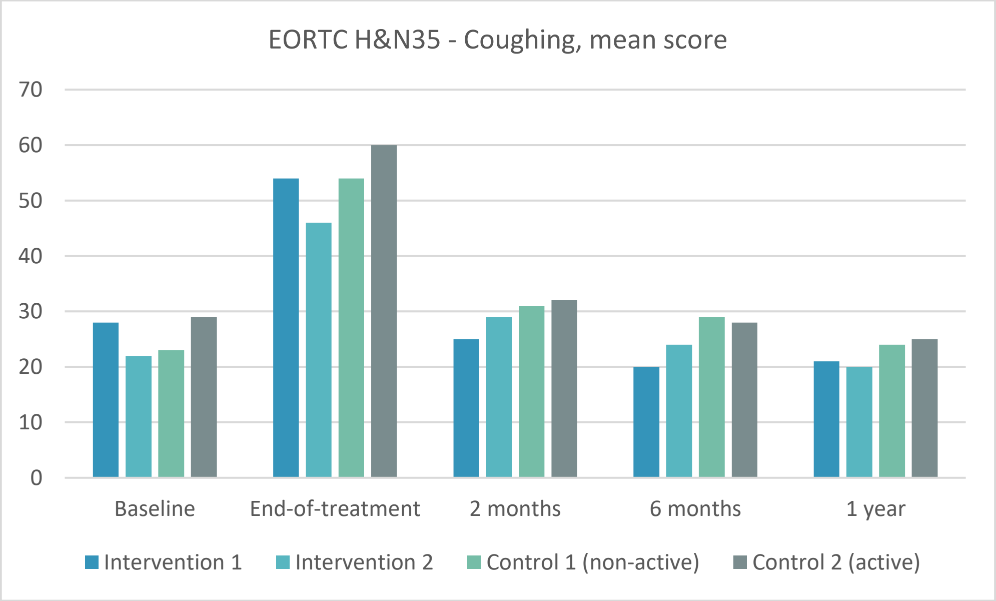

Online resource 4: Raw data graphs on four group

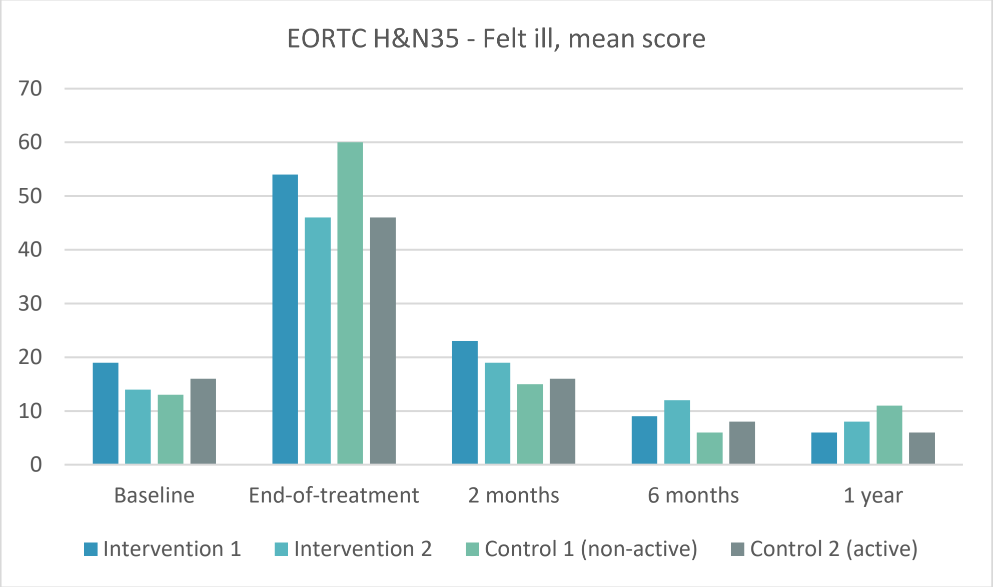

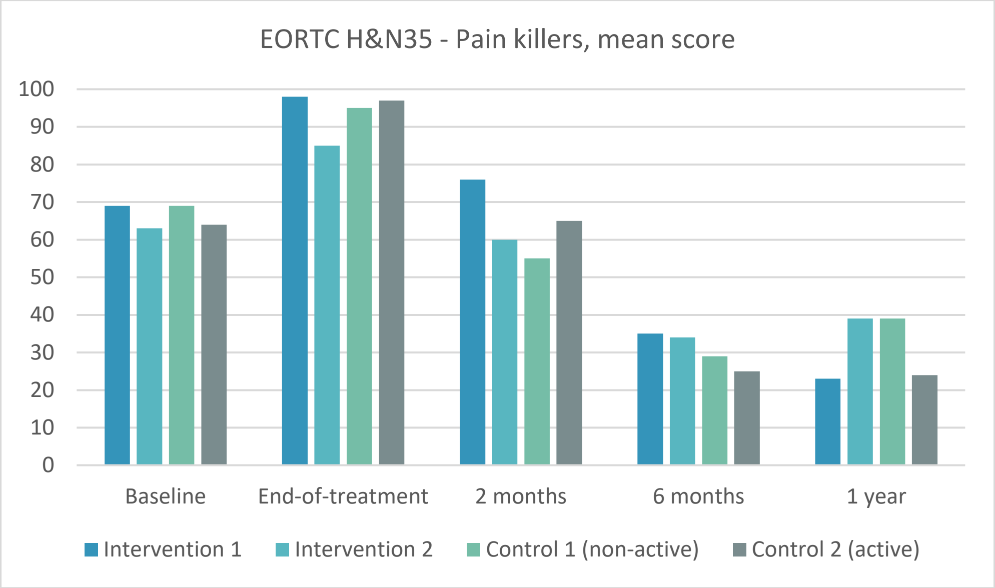

Article title: Swallowing exercises during head and neck cancer treatment – results of a randomized trial

Journal: *Dysphagia* .

Authors: Hajdú SF, Wessel I, Dalton SO, Eskildsen, SJ, Johansen C.

Corresponding author: Sara Fredslund Hajdú, dept of occupational therapy and physiotherapy, Copenhagen University Hospital Rigshospitalet, Denmark & Cancer Late Effects Research Unit (CASTLE), Department of Oncology, Copenhagen University Hospital Rigshospitalet, Denmark. sara.fredslund.hajdu@regionh.dk
